# Effectiveness of a School- and Primary Care–Based HPV Vaccination Intervention: The PrevHPV Cluster Randomized Trial

**DOI:** 10.1001/jamanetworkopen.2024.11938

**Published:** 2024-05-23

**Authors:** Nathalie Thilly, Morgane Michel, Maïa Simon, Aurélie Bocquier, Amandine Gagneux-Brunon, Aurélie Gauchet, Serge Gilberg, Anne-Sophie Le Duc-Banaszuk, Sébastien Bruel, Judith E. Mueller, Bruno Giraudeau, Karine Chevreul

**Affiliations:** 1 Université de Lorraine, APEMAC, Nancy, France; 2 Département Méthodologie, Promotion, Investigation, Université de Lorraine, CHRU-Nancy, Nancy, France; 3 Université Paris Cité, ECEVE, UMR 1123, Inserm, Paris, France; 4 Unité d’épidémiologie clinique, Assistance Publique-Hôpitaux de Paris, Hôpital Robert Debré, Paris, France; 5 CIC-1408 Vaccinologie, CHU de Saint-Etienne, Saint-Priest-en-Jarez, France; 6 Centre International de Recherche en Infectiologie, Team GIMAP, Univ Lyon, Université Jean Monnet, Université Claude Bernard Lyon 1, Inserm, U1111, Lyon, France; 7 Institut Presage Chaire Prevacci, Université Jean Monnet, Saint-Priest-en-Jarez, France; 8 Université Savoie Mont Blanc, Université Grenoble Alpes, LIP/PC2S, Grenoble, France; 9 Department of General Practice, Université Paris Cité, Paris, France; 10 Centre Régional de Coordination des Dépistages des cancers-Pays de la Loire, Angers, France; 11 Department of General Practice, Jacques Lisfranc Faculty of Medicine, Université Saint-Etienne-Lyon, Saint-Etienne, France; 12 Health, Systemic, Process UR 4129 Research Unit, Université Claude Bernard Lyon 1, Lyon, France; 13 Emerging Disease Epidemiology Unit, Institut Pasteur, Université Paris Cité, Paris, France; 14 Université de Rennes, EHESP, CNRS, Inserm, Arènes - UMR 6051, RSMS (Recherche sur les Services et Management en Santé) - U 1309, Rennes, France; 15 Université de Tours, Université de Nantes, SPHERE U1246, Inserm, Tours, France; 16 Inserm CIC 1415, CHRU de Tours, Tours, France

## Abstract

**Importance:**

The human papillomavirus (HPV) vaccine is safe and effective, yet vaccination coverage remains below public health objectives in many countries.

**Objective:**

To examine the effectiveness of a 3-component intervention on HPV vaccination coverage among adolescents aged 11 to 14 years 2 months after the intervention ended, each component being applied alone or in combination.

**Design, Setting, and Participants:**

A cluster randomized trial with incomplete factorial design (PrevHPV) was conducted between July 1, 2021, and April 30, 2022, in French municipalities receiving 0, 1, 2, or 3 components of the intervention. Randomization was stratified by school district and municipalities’ socioeconomic level. Analyses were carried out on 11- to 14-year-old adolescents living in all participating municipalities, regardless of what had been implemented.

**Intervention:**

The PrevHPV intervention had 3 components: (1) educating and motivating 11- to 14-year-old adolescents in middle schools, along with their parents; (2) training general practitioners (GPs) on up-to-date HPV information and motivational interviewing techniques; and (3) free HPV vaccination at school.

**Main Outcomes and Measures:**

The primary outcome was HPV vaccination coverage (≥1 dose) 2 months after the intervention ended among 11- to 14-year-old adolescents living in participating municipalities, based on the French national reimbursement database and data collected during the trial in groups randomized to implement at-school vaccination.

**Results:**

A total of 91 municipalities comprising 30 739 adolescents aged 11 to 14 years (15 876 boys and 14 863 girls) were included and analyzed. Half the municipalities were in the 2 lowest socioeconomic quintiles and access to GPs was poor in more than two-thirds of the municipalities. Thirty-eight of 61 schools (62.3%) implemented actions and 26 of 45 municipalities (57.8%) had at least 1 trained GP. The median vaccination coverage increased by 4.0 percentage points (IQR, 2.0-7.3 percentage points) to 14.2 percentage points (IQR, 9.1-17.3 percentage points) at 2 months. At-school vaccination significantly increased vaccination coverage (5.50 percentage points [95% CI, 3.13-7.88 percentage points]) while no effect was observed for adolescents’ education and motivation (−0.08 percentage points [95% CI, −2.54 to 2.39 percentage points]) and GPs’ training (−1.46 percentage points [95% CI, −3.44 to 0.53 percentage points]). Subgroup analyses found a significant interaction between at-school vaccination and access to GPs, with a higher effect when access was poor (8.62 percentage points [95% CI, 5.37-11.86 percentage points] vs 2.13 percentage points [95% CI, −1.25 to 5.50 percentage points]; *P* = .007 for interaction).

**Conclusions and Relevance:**

In this cluster randomized trial, within the context of the late COVID-19 pandemic period and limited school and GP participation, at-school HPV vaccination significantly increased vaccination coverage. The trial did not show a significant effect for training GPs and education and motivation, although it may be observed after more time has elapsed after the intervention.

**Trial Registration:**

ClinicalTrials.gov Identifier: NCT04945655

## Key Points

**Question:**

What is the effectiveness of a school- and primary care–based multicomponent intervention on human papillomavirus (HPV) vaccination coverage?

**Findings:**

This cluster randomized trial carried out in 91 municipalities found that free HPV vaccination on school premises was the only component that consistently and significantly increased vaccination coverage 2 months after the end of the intervention. No significant effect was found of training general practitioners and educating and motivating adolescents and their parents.

**Meaning:**

This study suggests that at-school vaccination is a useful tool to increase HPV vaccination coverage.

## Teaser

This cluster randomized trial examines the effectiveness of a school- and primary care–based multicomponent intervention to increase human papillomavirus vaccination coverage among children aged 11 to 14 years.

## Introduction

Human papillomavirus (HPV) infection is the most common viral sexually transmitted infection, and approximately 85% of sexually active people will get infected in their lifetime.^1,2,3^ Although most HPV infections resolve spontaneously, persistent infection with high-risk HPV types can cause cancer,^1^ with 690 000 new cancer cases being attributable to HPV in 2018 worldwide.^4^ As a result, HPV is a major public health concern.

Most high-income countries have recommended HPV vaccination for preteens and adolescents,^5,6,7,8^ as it is the only effective form of primary prevention against HPV infection. The vaccine is both safe and effective,^1,9,10,11,12^ including at reducing the risk of invasive cervical cancer,^13,14^ through an individual direct effect but also an indirect herd protection effect.^15,16^

However, vaccination coverage (VC) remains lower than expected in many countries.^5,6^ In France, where HPV vaccination has been recommended for all adolescents aged 11 to 14 years since 2021 after being initially recommended only for girls since 2007, with 2 injections and a catch-up of 3 injections up to 19 years of age,^17^ full VC was 41.5% among girls aged 16 years and 8.5% among boys aged 16 years in 2022.^18^

It is therefore of great importance to identify effective means of increasing HPV VC. To date, too few interventions have included a multilevel approach, despite research showing its effectiveness, or indeed have reported their effect on VC.^19^

The PrevHPV research program was launched in 2018 to improve HPV acceptability and VC in France. It used the Integrated Behavior Change Model as the theoretical background.^20^ First, published evidence on facilitators and barriers to the uptake of HPV vaccination, and on existing interventions to improve vaccination in general and for HPV in particular among adolescents helped identify the framework of a 3-component intervention: (1) educating and motivating adolescents in middle schools; (2) training general practitioners (GPs) on up-to-date HPV information and motivational interviewing techniques; and (3) free HPV vaccination at school. A diagnostic phase was then carried out to identify—among others—knowledge, beliefs, barriers, and preferences around HPV vaccination and the sociodemographic factors associated with each,^21,22,23,24^ to define the exact content of each component to appropriately address vaccine hesitancy in our population. The final version of the PrevHPV multicomponent intervention was constructed with the target populations to fit to all French adolescents, regardless of their sociodemographic characteristics. It was then assessed in an experimental phase. The objective of the present study is to evaluate the effectiveness of the PrevHPV intervention on VC 2 months after the end of the intervention, each of its components being applied alone or in combination.

## Methods

### Study Design

A pragmatic, open-label, cluster randomized trial using an incomplete factorial design was carried out between July 1, 2021, and April 30, 2022, to evaluate the effectiveness of the PrevHPV intervention under routine conditions. It was granted approval by the French Ethics Committee Comité de Protection des Personnes–CPP Sud-Est VI. No individual consent was required for this type of research because in accordance with French law, this type of study is considered not to pose any risk to participants and as such investigators are required to make sure that participants do not oppose participation, and not that they consent. All participants (adolescents, parents, and GPs) were informed of their rights, in particular, not to participate or to oppose the collection of data concerning them. The Consolidated Standards of Reporting Trials (CONSORT) reporting guideline, statement, and its relevant extensions were used as a reporting guide for the present article.^26^

The study protocol has been published previously and can be found in Supplement 1.^25^ In brief, the cluster was the municipality. Municipalities were eligible if (1) they were located in 1 of 14 school districts (ie, close to two-thirds of all districts), selected to ensure a diversity of geographical, demographic, and socioeconomic profiles; (2) there was only 1 middle school in the municipality; and (3) at least two-thirds of 11-to 14-year-old adolescents living in that municipality attended the middle school. A group of eligible municipalities was selected by random sampling. Only municipalities whose middle school agreed to participate were included in the study and randomized. At the individual level, all GPs practicing in participating municipalities were eligible unless they practiced alternative medicine (eg, homeopathy or acupuncture) exclusively, planned to stop their professional activity in the municipality before the end of 2021, or had no internet access. Adolescents were eligible to be vaccinated at school if they had never been vaccinated against HPV, were 11 years of age or older, had no contraindication, and if their parents had given written consent for them to be vaccinated.

The factorial design allows for the evaluation of multiple factors simultaneously—here, the different components of the intervention, explored alone and in combination. Under a full factorial design, all possible combinations of the components are assessed; however, our design was incomplete because at-school vaccination was never carried out without prior education and motivation of adolescents to allow for an informed decision, as there is suboptimal knowledge in France about HPV and its vaccine.^27^

Municipalities were randomized all at once into 1 of 6 groups (Figure), using block randomization (block size = 6) stratified by school district and municipalities’ socioeconomic level—assessed by a French ecological index, the French Deprivation Index (FDep).^28^ Randomization was computer generated with SAS software, version 9.4 (SAS Institute Inc), by a senior researcher of the PrevHPV Study Group (N.T.) not involved in the selection process of the municipalities.

**Figure.**
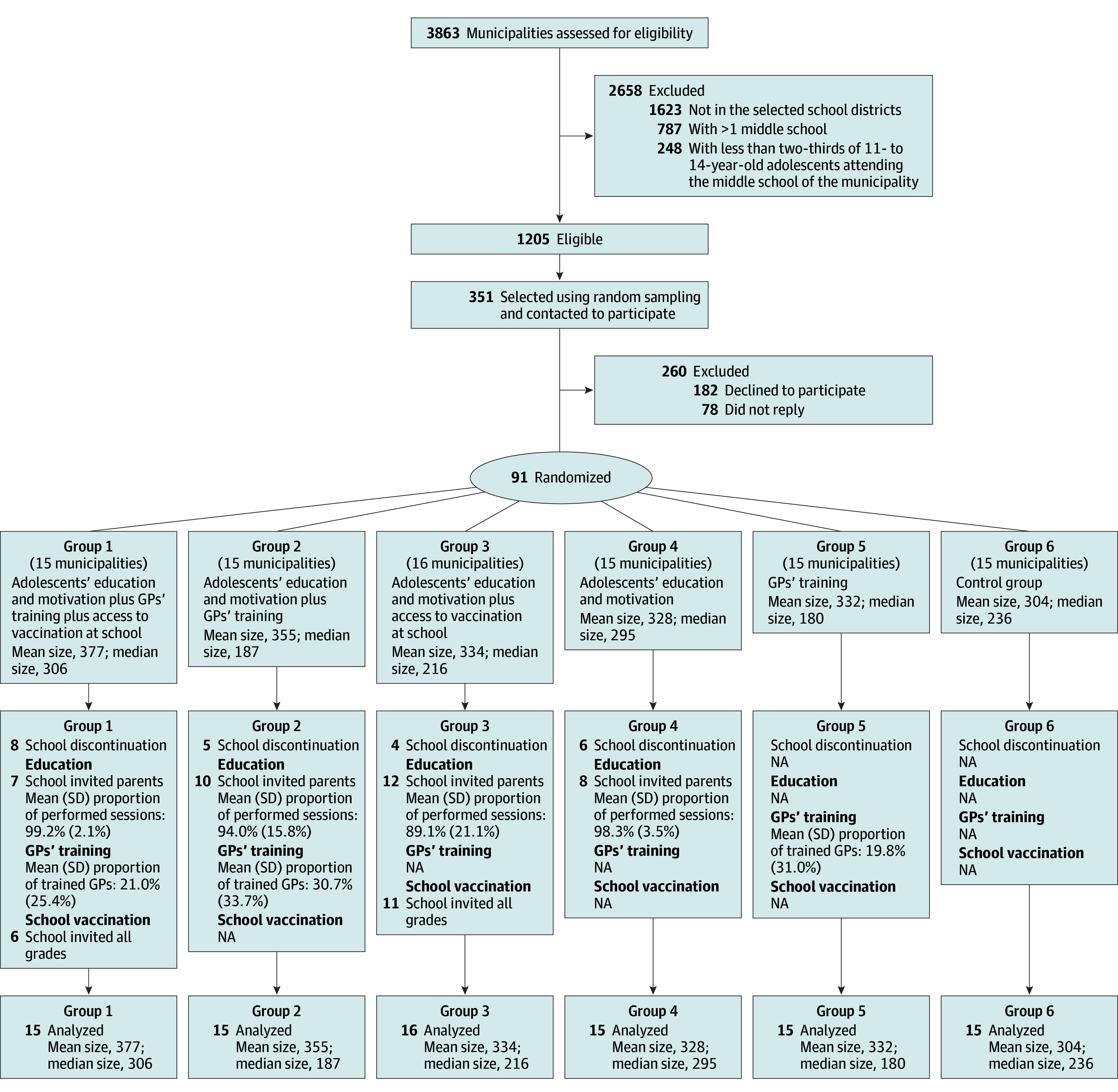
Flowchart of the Municipalities’ Selection and Group Allocation GP indicates general practitioner; NA, not applicable.

### Description of the Intervention

The PrevHPV intervention was composed of 3 components. Details about its development and content have been published previously.^29^

Component 1 (adolescents’ education and motivation) was carried out in middle schools. Parents were first invited by the school to a webconference to receive information on HPV infection and vaccination (from December 2021 to January 2022). Pupils in grades 8 and 9 (13-14 years of age) then attended 2 educational group sessions on those topics during school hours (from January to March 2022). Each session used participatory learning and e-health tools (videos, serious game) developed by the PrevHPV program.

Component 2 (GPs’ training) offered individual e-learning training to eligible GPs on a voluntary basis (available from January to April 2022). It included up-to-date information on HPV infection and vaccination, an introduction to motivational interviewing techniques, and a decision aid tool developed by the PrevHPV program, provided to GPs in attendance. Its goal was to help hesitant parents and adolescents come to a shared decision regarding HPV vaccination.

Component 3 (HPV vaccination at school) consisted in offering free vaccination on school premises on specific days (from March to April 2022 [ie, after the educational group sessions had been completed]). Vaccines were administered by health professionals from local vaccination centers to eligible adolescents.

### Outcome

The primary outcome was HPV VC (≥1 dose) among adolescents aged 11 to 14 years who lived in participating municipalities, 2 months after the end of the intervention (June 30, 2022).

### Data Sources

Two data sources were used. The French national reimbursement database (Système National des Données de Santé [SNDS]), which contains pseudonymized data on all health care consumption reimbursed by the statutory health insurance for more than 99% of the French population,^30^ provided beneficiaries’ postcode of residence (ie, their municipality) and information on vaccines delivered in community pharmacies for vaccinations prescribed by GPs and other authorized health care professionals, as well as the age and sex of beneficiaries.

Data collected during the trial provided information on the number of adolescents vaccinated at school (not recorded in the SNDS database) in the groups randomized to implement this component, along with the child’s age, sex, and municipality of residence. The total number of vaccinated adolescents was obtained by summing vaccines recorded in each data source.

### Statistical Analysis

The sample size calculation assumed a mean number of pupils per middle school of 466, with a coefficient of variation of 0.5 (based on data from the National Education Ministry). It was performed in 2019, when HPV VC (≥1 dose) among French adolescents in the age group of 11 to 14 years was estimated at 8% (due to boys having a 0% VC at the time). A 10–percentage point increase in VC between 2 groups was expected, whatever the intervention component. Considering an intraclass correlation of 0.05, a sample of 15 municipalities per group (ie, 90 municipalities in total) was needed to achieve a 90% power considering a 5% α risk. The calculation was carried out using the Shiny app calculator.^31^

The analysis was carried out on all municipalities included in the trial, regardless of what was implemented. A descriptive analysis of their characteristics was first carried out by group using numbers and percentages for categorical variables and median (IQR) values for continuous variables. Vaccination coverage was described at baseline (June 30, 2021) and 2 months after the end of the intervention (June 30, 2022), along with its evolution in percentage points. The effectiveness of each component was assessed using a cluster-level analysis in which each cluster was weighted by its size (number of adolescents aged 11-14 years living in the municipality according to the SNDS database). Analysis units were therefore independent, which led us to fit a linear model including 3 fixed effects (1 per component), adjusted on baseline HPV VC (derived from the SNDS database) and their associated 2-level and 3-level interactions terms. A likelihood ratio test was used to compare this model and the embedded model without any interaction term. A nonsignificant likelihood ratio test was considered as an argument to discard all interaction terms from subsequent analyses.

A priori–specified subgroup analyses were carried out based on (1) sex, because the recommendation to vaccinate boys against HPV was still recent, and (2) municipalities’ socioeconomic level, because socioeconomic disparities have been evidenced in HPV VC in France,^32^ and prevention programs may affect social inequalities in health.^33^ For sex, because it is an individual-level variable, we reran the main analysis considering 2 distinct outcomes (ie, 1 outcome for each sex). For socioeconomic level—a cluster-level variable—municipalities were split into 2 groups: the 2 lowest quintiles based on the FDep’s national distribution (quintiles 4 and 5) vs the other 3 quintiles. Vaccination coverage was first calculated regardless of the intervention group, to evidence eventual social inequalities in our sample. A 2-sided Wilcoxon-Mann-Whitney test compared VC in the 2 groups, before and after the intervention. Then, the subgroup analysis was conducted by fitting a linear model considering the dichotomized FDep variable as a fixed effect and 3 interaction terms, one for each component of the intervention. We also refitted the linear model used for the main analysis after adding the continuous FDep variable as an adjustment variable.

Finally, an a posteriori subgroup analysis was performed based on access to GPs in the municipality using the local potential accessibility indicator,^34^ because the effectiveness of the intervention may affect or be affected by territorial inequalities in access to care.^32^ We used the same strategy as for the FDep, except the lowest quintiles corresponded to quintiles 1 and 2. All analyses were performed using SAS, version 9.4 (SAS Institute Inc). *P* < .05 was considered statistically significant.

## Results

### Characteristics of the Municipalities

Ninety-one municipalities were included and randomized all at once into 6 groups (Figure), comprising 30 739 adolescents aged 11 to 14 years (15 876 boys and 14 863 girls). The median number of 11- to 14-year-old adolescents living in those municipalities ranged from 180 (IQR, 68-571) to 306 (IQR, 192-390), reflecting chance imbalance (Table 1). Approximately half the municipalities belonged to the 2 lowest socioeconomic quintiles of French municipalities, and access to GPs was poor in more than two-thirds of the municipalities. Most middle schools were public, and their median number of pupils ranged from 370 (IQR, 303-602) to 506 (IQR, 248-600) (Table 1).

**Table 1.** Characteristics of the 91 Participating Municipalities

Characteristic	Group 1: at-school vaccination plus adolescents’ education and motivation plus GPs’ training (n = 15)	Group 2: adolescents’ education and motivation plus GPs’ training (n = 15)	Group 3: at-school vaccination plus adolescents’ education and motivation (n = 16)	Group 4: adolescents’ education and motivation (n = 15)	Group 5: GPs’ training (n = 15)	Group 6: control (n = 15)
**Sociodemographic characteristics of municipalities**						
No. of inhabitants, median (IQR)	4714 (2807 to 6782)	2902 (1327 to 10 793)	3958 (2293 to 5218)	4229 (2166 to 6166)	2336 (1080 to 9349)	3395 (2036 to 5962)
No. of adolescents aged 11-14 y, median (IQR)	306 (192 to 390)	187 (104 to 624)	216 (141 to 349)	295 (120 to 418)	180 (68 to 571)	236 (117 to 482)
Rural (vs urban), No. (%)	7 (46.7)	11 (73.3)	11 (68.7)	8 (53.3)	9 (60.0)	9 (60.0)
Socioeconomic level (FDep), median (IQR)	0.30 (−0.49 to 0.71)	0.32 (−0.17 to 1.04)	0.40 (−0.54 to 0.84)	0.33 (−0.38 to 1.09)	0.40 (−0.67 to 1.00)	0.52 (−0.80 to 1.01)
Highest, No. (%)^a^	8 (55.3)	8 (55.3)	6 (37.5)	8 (53.3)	7 (46.7)	7 (46.7)
Lowest, No. (%)^a^	7 (46.7)	7 (46.7)	10 (62.5)	7 (46.7)	8 (53.3)	8 (53.3)
**Characteristics of GPs**						
No. of GPs, median (IQR)	4 (3 to 7)	2 (1 to 5)	4 (2.5 to 5)	4 (2 to 8)	3 (2 to 7)	3 (2 to 6)
Access to GPs (LPA), median (IQR)	3.3 (2.6 to 3.7)	3.6 (2.5 to 4.2)	3.5 (2.4 to 4.3)	3.3 (2.9 to 3.7)	3.2 (2.8 to 3.5)	3.2 (2.7 to 4.5)
Least access, No. (%)^b^	9 (60.0)	7 (46.7)	8 (50.0)	10 (66.7)	12 (80.0)	8 (53.3)
Most access, No. (%)^b^	6 (40.0)	8 (53.3)	8 (50.0)	5 (33.3)	3 (20.0)	7 (46.7)
**Characteristics of middle schools**						
No. of pupils, median (IQR)	483 (338 to 643)	402 (232 to 470)	370 (303 to 602)	383 (200 to 594)	436 (268 to 620)	506 (248 to 600)
Public school (vs private), No. (%)	14 (93.3)	14 (93.3)	15 (93.7)	15 (100.0)	15 (100.0)	15 (100.0)

Abbreviations: FDep, French Deprivation Index (based on the percentage of blue-collar workers in the labor force, percentage of high school graduates in the population aged 15 years or older, unemployment rate, and median income per household); GP, general practitioner; LPA, local potential accessibility (based on the number of GPs, their activity, age of the population, and distance to GPs’ offices in the municipality).

^a^ Highest socioeconomic level defined as the first 3 quintiles based on the national distribution of the FDep variable and lowest socioeconomic level defined as the last 2 quintiles.

^b^ Least access defined as the first 2 quintiles based on the national distribution of the LPA variable and most access defined as the last 3 quintiles.

### Intervention Delivered

Between 25.0% (4 of 16) and 53.3% (8 of 15) of schools randomized to a school-based component dropped out of the study, while overall 62.3% of schools (38 of 61) implemented at least part of a component (Figure). The characteristics of municipalities whose school did and did not drop out were similar (eTable in Supplement 2). Among schools that implemented the intervention at least in part, all but 1 invited parents to the webconference, and the mean (SD) proportion of educational sessions carried out was between 89.1% (21.2%) and 99.2% (2.1%) (Figure). Most schools invited students in all eligible grades to be vaccinated, apart from 2 that invited only students from grades 8 and 9. In groups randomized to GPs’ training, 57.8% of municipalities (26 of 45) had at least 1 GP trained. The mean (SD) proportion of trained GPs was between 19.8% (31.0%) and 30.7% (33.7%).

### Effectiveness of the Intervention on VC

At baseline, the median VC was 13.6% (IQR, 10.2%-17.2%) among 11- to 14-year-old adolescents living in participating municipalities. The median VC varied between groups from 8.9% (IQR, 6.7%-14.5%) to 15.0% (IQR, 10.7%-19.4%) (Table 2).

**Table 2.** HPV Vaccination Coverage for 11- to 14-Year-Old Adolescents at Baseline and 2 Months After End of Intervention in Participating Municipalities^a^

Characteristic	Median (IQR) value					
	Group 1: at-school vaccination plus adolescents’ education and motivation plus GPs’ training (n = 15)	Group 2: adolescents’ education and motivation plus GPs’ training (n = 15)	Group 3: at-school vaccination plus adolescents’ education and motivation (n = 16)	Group 4: adolescents’ education and motivation (n = 15)	Group 5: GPs’ training (n = 15)	Group 6: control (n = 15)
**All adolescents aged 11-14 y**						
At baseline, %	11.7 (10.3 to 19.4)	12.0 (8.2 to 17.1)	14.8 (12.3 to 19.2)	15.0 (10.7 to 19.4)	8.9 (6.7 to 14.5)	13.8 (10.9 to 17.2)
At 2 mo, %	23.8 (12.7 to 37.1)	20.1 (12.2 to 24.8)	30.0 (24.3 to 34.1)	17.5 (12.8 to 27.3)	11.7 (9.1 to 24.4)	17.8 (14.0 to 24.1)
Difference, percentage points	8.1 (2.7 to 20.3)	5.1 (3.0 to 7.9)	14.2 (9.1 to 17.3)	6.6 (2.0 to 8.7)	4.0 (2.0 to 7.3)	4.6 (1.9 to 7.4)
**Girls aged 11-14 y**						
At baseline, %	22.7 (16.2 to 31.0)	22.2 (15.8 to 28.7)	25.4 (21.5 to 34.6)	23.7 (19.3 to 29.6)	16.0 (11.8 to 24.7)	27.3 (16.4 to 31.7)
At 2 mo, %	37.7 (21.2 to 46.2)	27.0 (18.5 to 33.3)	38.4 (31.6 to 42.7)	31.0 (22.6 to 35.8)	20.4 (15.1 to 33.3)	29.1 (25.8 to 34.6)
Difference, percentage points	7.4 (2.6 to 19.8)	3.7 (2.0 to 6.0)	10.3 (4.6 to 13.0)	5.5 (−0.3 to 9.0)	3.4 (0.3 to 6.7)	3.3 (1.3 to 10.4)
**Boys aged 11-14 y**						
At baseline, %	3.0 (1.0 to 7.3)	4.0 (2.6 to 7.4)	3.7 (2.3 to 6.6)	5.2 (1.9 to 7.7)	1.8 (0 to 4.7)	3.4 (2.2 to 5.9)
At 2 mo, %	13.7 (4.8 to 25.8)	11.5 (5.2 to 18.1)	22.0 (17.2 to 27.4)	11.2 (5.2 to 15.4)	7.4 (3.4 to 10.6)	7.2 (5.5 to 13.8)
Difference, percentage points	8.9 (2.7 to 22.9)	7.7 (3.0 to 10.8)	17.1 (12.1 to 22.6)	6.0 (3.3 to 8.6)	5.1 (1.1 to 10.0)	4.2 (2.2 to 6.9)
**All adolescents aged 11-14 y, by socioeconomic level of the municipality (FDep)**						
Highest^b^						
At baseline, %	13.6 (10.9 to 19.9)	16.1 (11.3 to 24.1)	14.5 (12.3 to 15.1)	16.7 (13.7 to 20.2)	14.2 (8.9 to 21.7)	13.8 (10.9 to 16.3)
At 2 mo, %	23.4 (16.3 to 34.5)	22.5 (16.2 to 29.8)	28.9 (18.5 to 38.6)	23.7 (16.9 to 28.1)	18.5 (13.9 to 24.7)	17.8 (14.0 to 18.5)
Difference, percentage points	7.7 (3.6 to 14.4)	5.2 (3.6 to 8.3)	12.7 (6.4 to 17.6)	8.2 (3.9 to 8.8)	4.1 (3.0 to 9.8)	3.1 (1.9 to 4.6)
Lowest^b^						
At baseline, %	11.7 (7.8 to 16.1)	11.5 (7.8 to 14.1)	15.9 (12.4 to 20.0)	11.0 (9.7 to 15.4)	7.1 (6.5 to 9.0)	14.4 (10.6 to 17.4)
At 2 mo, %	30.2 (12.2 to 37.1)	19.4 (10.8 to 20.5)	30.0 (26.6 to 32.6)	16.4 (11.9 to 21.6)	10.6 (8.7 to 11.4)	20.5 (13.7 to 26.5)
Difference, percentage points	14.1 (0.7 to 21.3)	5.0 (3.0 to 7.8)	14.3 (9.5 to 17.2)	2.2 (2.0 to 6.7)	2.7 (0.6 to 6.4)	7.1 (2.6 to 11.8)
**All adolescents aged 11-14 y, by access to general practitioners in the municipality (LPA)**						
Least access^c^						
At baseline, %	11.7 (10.7 to 20.3)	11.5 (7.8 to 15.0)	16.1 (12.2 to 20.6)	15.0 (11.0 to 18.4)	8.1 (6.5 to 9.4)	12.9 (10.7 to 15.1)
At 2 mo, %	28.4 (12.7 to 40.5)	20.1 (12.2 to 20.5)	32.6 (27.0 to 37.1)	17.2 (11.9 to 27.1)	10.9 (8.7 to 15.0)	18.0 (16.5 to 21.2)
Difference, percentage points	8.1 (2.7 to 21.3)	5.1 (3.0 to 7.8)	16.5 (10.9 to 21.1)	4.4 (2.0 to 8.7)	3.7 (1.5 to 7.3)	4.5 (2.1 to 7.2)
Most access^c^						
At baseline, %	13.7 (10.3 to 16.1)	14.5 (9.4 to 19.9)	14.1 (12.3 to 16.8)	12.6 (10.7 to 19.6)	21.7 (14.2 to 33.8)	16.9 (10.9 to 17.7)
At 2 mo, %	23.4 (18.7 to 30.2)	19.3 (13.4 to 28.2)	26.9 (21.9 to 30.5)	20.3 (17.0 to 27.3)	24.7 (18.4 to 46.6)	14.6 (12.8 to 28.1)
Difference, percentage points	7.9 (4.4 to 14.1)	5.2 (3.5 to 8.3)	11.6 (7.6 to 15.6)	7.8 (5.8 to 8.6)	4.1 (3.0 to 12.8)	5.5 (−0.0 to 10.9)

Abbreviations: FDep, French Deprivation Index; GP, general practitioner; HPV, human papillomavirus; LPA, local potential accessibility.

^a^ Vaccination coverage = number of adolescents aged 11 to 14 years who had received at least 1 dose of HPV vaccine and who lived in a participating municipality at the time of the evaluation (June 30, 2021, for baseline; and June 30, 2022, for 2 months) divided by the total number of adolescents in that age group living in the municipality at that time.

^b^ Highest socioeconomic level defined as the first 3 quintiles based on the national distribution of the FDep variable and lowest socioeconomic level defined as the last 2 quintiles.

^c^ Least access defined as the first 2 quintiles based on the national distribution of the LPA variable and most access defined as the last 3 quintiles.

Two months after the end of the intervention, the median VC increased in all groups, from 4.0 percentage points (IQR, 2.0-7.3 percentage points) to 14.2 percentage points (IQR, 9.1-17.3 percentage points). The increase was highest in the 2 groups with at-school vaccination (8.1 percentage points [IQR, 2.7-20.3 percentage points] and 14.2 percentage points [IQR, 9.1-17.3 percentage points] compared with baseline), while in the control group, the median VC increased from 13.8% (IQR, 10.9%-17.2%) to 17.8% (IQR, 14.0%-24.1%) (Table 2).

At-school vaccination was shown to significantly increase VC in the regression model (5.50 percentage points [95% CI, 3.13-7.88 percentage points]) (Table 3). We did not observe any significant effect for adolescents’ education and motivation (−0.08 percentage points [95% CI, −2.54 to 2.39 percentage points]) and GPs’ training (−1.46 percentage points [95% CI, −3.44 to 0.53 percentage points]). The likelihood ratio test that globally assessed the significance of the interaction terms was not significant (*P* = .44).

**Table 3.** Effectiveness of Each Component of Intervention on HPV Vaccination Coverage at 2 Months in 11- to 14-Year-Old Adolescents

Component	Estimate (95% CI), percentage points^a^	*P* value
**Adolescents’ education and motivation**		
Adolescents aged 11-14 y	−0.08 (−2.54 to 2.39)	.95
Girls	−0.88 (−3.63 to 1.87)	.53
Boys	−0.08 (−3.00 to 2.84)	.96
Adjusting for socioeconomic level of the municipality	−0.14 (−2.62 to 2.34)	.91
Adjusting for access to general practitioners in the municipality	−0.08 (−2.55 to 2.93)	.95
**General practitioners’ training**		
Adolescents aged 11-14 y	−1.46 (−3.44 to 0.53)	.15
Girls	−1.83 (−4.05 to 0.40)	.11
Boys	−1.71 (−4.00 to 0.57)	.14
Adjusting for socioeconomic level of the municipality	−1.42 (−3.41 to 0.57)	.16
Adjusting for access to general practitioners in the municipality	−1.46 (−3.44 to 0.53)	.15
**Vaccination at school**		
Adolescents aged 11-14 y	5.50 (3.13 to 7.88)	<.001
Girls	5.51 (2.85 to 8.17)	<.001
Boys	5.74 (2.95 to 8.52)	<.001
Adjusting for socioeconomic level of the municipality	5.58 (3.17 to 7.99)	<.001
Adjusting for access to general practitioners in the municipality	5.51 (3.13 to 7.88)	<.001

Abbreviation: HPV, human papillomavirus.

^a^ Regression coefficient obtained from the regression model adjusted for baseline vaccination coverage.

### Effectiveness According to Sex

At baseline, the median VC varied between 16.0% (IQR, 11.8%-24.7%) and 27.3% (IQR, 16.4%-31.7%) among girls and between 1.8% (IQR, 0%-4.7%) and 5.2% (IQR, 1.9%-7.7%) among boys (Table 2). The increase in the median VC at 2 months was higher among boys in all groups, especially in the 2 groups with at-school vaccination. In the control group, the median VC increased from 3.4% (IQR, 2.2%-5.9%) to 7.2% (IQR, 5.5%-13.8%) among boys and from 27.3% (IQR, 16.4%-31.7%) to 29.1% (IQR, 25.8%-34.6%) among girls.

In the regression model, at-school vaccination was shown to have an effectiveness of similar magnitude among both sexes after adjusting for baseline VC: 5.51 percentage points for girls (95% CI, 2.85-8.17 percentage points) and 5.74 percentage points for boys (95% CI, 2.95-8.52 percentage points) (Table 3). We did not observe any significant effect for the other components.

### Effectiveness According to Municipalities’ Socioeconomic Level

At baseline, a lower median VC was observed in the municipalities with the lowest socioeconomic level compared with those with the highest socioeconomic level (11.5% [IQR, 8.2%-16.9%] vs 14.7% [IQR, 11.2%-19.5%]; *P* = .02). This difference was no longer significant at 2 months (20.1% [IQR, 11.7%-28.5%] vs 20.2% [IQR, 16.1%-28.3%]; *P* = .32).

The median increase in VC was highest in the municipalities with the lowest socioeconomic level in the 2 groups where at-school vaccination was implemented (Table 2). In the control group, the median VC increased from 13.8% (IQR, 10.9%-16.3%) to 17.8% (IQR, 14.0%-18.5%) in the municipalities with the highest socioeconomic level and from 14.4% (IQR, 10.6%-17.4%) to 20.5% (IQR, 13.7%-26.5%) in the municipalities with the lowest socioeconomic level.

The effect of the at-school vaccination component found in the main regression model remained significant and of the same magnitude when adjusting for the socioeconomic level of the municipality (5.58 percentage points [95% CI, 3.17-7.99 percentage points]) (Table 3). It was estimated at 6.12 percentage points (95% CI, 2.70-9.54 percentage points) vs 4.98 percentage points (95% CI, 1.65-8.31 percentage points) in the municipalities with the lowest socioeconomic level compared with the municipalities with the highest socioeconomic level, although this difference was not significant (*P* = .64) (Table 4).

**Table 4.** Effectiveness of Each Component of Intervention on HPV Vaccination Coverage at 2 Months in 11- to 14-Year-Old Adolescents When Including an Interaction Term With Socioeconomic Level or Access to General Practitioners in the Municipalities

Component	Estimate (95% CI), percentage points^a^	*P* value	*P* value for interaction
**By socioeconomic level of the municipality (FDep)**			
Adolescents’ education and motivation			
Highest socioeconomic level^b^	1.30 (−2.07 to 4.68)	.45	.22
Lowest socioeconomic level^b^	−1.82 (−5.42 to 1.78)	.32	
General practitioners’ training			
Highest socioeconomic level^b^	−1.00 (−3.66 to 1.67)	.46	.68
Lowest socioeconomic level^b^	−1.85 (−4.92 to 1.22)	.24	
Vaccination at school			
Highest socioeconomic level^b^	4.98 (1.65 to 8.31)	.003	.64
Lowest socioeconomic level^b^	6.12 (2.70 to 9.54)	<.001	
**By access to general practitioners in the municipality (LPA)**			
Adolescents’ education and motivation			
Least access^c^	−0.82 (−3.91 to 2.27)	.60	.33
Most access^c^	1.61 (−2.15 to 5.36)	.30	
General practitioners’ training			
Least access^c^	−1.06 (−3.70 to 1.57)	.43	.34
Most access^c^	−2.96 (−5.82 to −0.10)	.04	
Vaccination at school			
Least access^c^	8.62 (5.37 to 11.86)	<.001	.007
Most access^c^	2.13 (−1.25 to 5.50)	.22	

Abbreviations: FDep, French Deprivation Index; HPV, human papillomavirus; LPA, local potential accessibility.

^a^ Regression coefficient obtained from the regression model adjusted for baseline vaccination coverage.

^b^ Highest socioeconomic level defined as the first 3 quintiles based on the national distribution of the FDep variable and lowest socioeconomic level defined as the last 2 quintiles.

^c^ Least access defined as the first 2 quintiles based on the national distribution of the LPA variable and most access defined as the last 3 quintiles.

### Effectiveness According to Municipalities’ Access to GPs

Although the median baseline VC was lower in municipalities with poor access to GPs than in municipalities with most access to GPs (11.8% [IQR, 9.1%-15.4%] vs 14.6% [IQR, 10.9%-18.4%]), the difference was not significant (*P* = .06). The same was observed at 2 months (18.4% [IQR, 11.9%-28.1%] vs 23.8% [IQR, 15.9%-28.5%]; *P* = .16).

The increase in the median VC was highest in the groups with at-school vaccination in municipalities with the least access (Table 2). In the control group, the median VC increased from 12.9% (IQR, 10.7%-15.1%) to 18.0% (IQR, 16.5%-21.2%) in municipalities with the least access and decreased from 16.9% (IQR, 10.9%-17.7%) to 14.6% (IQR, 12.8%-28.1%) in municipalities with the most access.

The effect of at-school vaccination found in the main regression model was still significant when adjusting for access to GPs (5.51 percentage points [95% CI, 3.13-7.88 percentage points]) (Table 3). In addition, there was a significant interaction between access to GPs and at-school vaccination, with a higher effect of the component in municipalities with the least access (8.62 percentage points [95% CI, 5.37-11.86 percentage points] vs 2.13 percentage points [95% CI, −1.25 to 5.50 percentage points]; *P* = .007) (Table 4).

## Discussion

In this cluster randomized trial, of the 3 components of the PrevHPV intervention, at-school vaccination led to a significant 5.50–percentage point increase in VC among 11- to 14-year-old adolescents 2 months after the end of the intervention. Its effectiveness was not significantly different between sexes. Municipalities’ socioeconomic level did not modify the effect on VC, but there was a higher effect of at-school vaccination among adolescents living in municipalities with poor access to GPs.

Our results align with the literature. At-school vaccination has long been shown to increase VC, leading many countries, such as the UK, Spain, and Scandinavian countries, to implement it.^6^ In France, local experimentations found it to be effective,^35,36^ and the PrevHPV national trial reinforces its effectiveness and feasibility. Although the increase reported in PrevHPV may appear modest, one-third of middle schools dropped out before implementing the intervention, so its effect is likely underestimated due to it being calculated on all randomized municipalities regardless of what was truly implemented.

Regarding the effect of training GPs, the literature appears more mixed, with some studies reporting an increase in VC^37,38^ and others not.^39,40^ This discrepancy may be due to differences in interventions (eg, education session, integrated reminder in health records, and decision-aid tools), making it difficult to compare findings among themselves and with our own. In addition, there was limited GP participation in our study: close to half of municipalities had no GP trained, which could explain why we did not find a significant effect for this component. In addition to lack of time, GPs’ own vaccine hesitancy may have influenced their participation.^41,42^

Similarly, we did not find that the education and motivation component for adolescents had a significant effect on VC; the literature tends to show that while informing people increases knowledge and is therefore necessary, it does not lead to significant increases in VC.^43,44,45^ In both cases, there may also be a financial barrier because, while at-school vaccination was free, families of adolescents vaccinated by GPs had to pay a copayment if they had no complementary health insurance, which is more often the case among low-income households.^46^

Studies have reported social inequalities in HPV vaccination, with localities with a lower socioeconomic level having lower HPV VC,^32^ which is consistent with our findings at baseline. This difference was no longer significant at 2 months; however, we did not find any significant interaction between the intervention and municipalities’ socioeconomic level, which may be due to a lack of power. Population prevention strategies may also negatively affect social health inequalities,^33^ as people with lower socioeconomic status may participate less in prevention programs,^47,48^ but we did not find any evidence for this with PrevHPV.

Regarding territorial inequalities, a French study found that HPV vaccine initiation increased with the number of contacts with GPs or gynecologists,^32^ and in territories with poorer access, vaccination rates were lower. We showed a significantly higher effect of at-school vaccination when access to GPs was poor, so this component may be a useful tool to mitigate territorial inequalities by providing vaccination regardless of GPs’ local availability.

To our knowledge, our study is the first to evaluate a multicomponent intervention aimed at improving HPV VC in France. It was coconstructed with its target populations and its evaluation was carried out with a high level of evidence thanks to its cluster randomized design. Its factorial design also allowed us to evaluate the effectiveness of its 3 components. Finally, all randomized municipalities were analyzed, and despite a low participation or high dropout rate, at-school vaccination was still found to significantly increase VC.

### Limitations

Our study has some limitations. The implementation started in July 2021 (for GPs) and October 2021 (for schools), after 18 months of COVID-19–related mobilization. Schools were dealing with contact tracing and COVID-19 vaccination, which led to many schools electing to drop out or being unable to implement the full intervention. Likewise, many health care professionals were overworked after the pandemic,^49^ which led to difficulties in involving GPs. Our findings are therefore likely to have been affected by the context, and an analysis considering the intervention dose actually delivered at the municipality level will be undertaken to enrich our results.

Because the factorial design was incomplete, it was not possible to evaluate the interaction between at-school vaccination and education and motivation of adolescents, as the former was never carried out without the latter. As such, the effect of at-school vaccination is based on the hypothesis that there is no interaction between the 2, which cannot be verified. This hypothesis may have led to a misestimation of the effect of this component delivered by itself. Its effect should also always be interpreted by considering it in adjunction to the effect of the education and motivation component.

Another limitation is that only municipalities with 1 middle school in their territory were included. Municipalities are the smallest geographical unit available in the SNDS database and doing otherwise would have required that all schools in a municipality agree to participate, which was less feasible. As a result, larger French municipalities were de facto excluded and participating municipalities are mostly small and rural, which raises the question of the generalizability of our results. However, the effect of that choice is likely to be minimal, as a previous study found that HPV vaccine uptake did not vary significantly according to the number of inhabitants in a municipality.^32^

Regarding the primary outcome, it is possible that the time horizon was too short to highlight the benefits of the education and motivation and GPs’ training components. They require that parents schedule 2 visits with their GP (1 for the vaccine prescription and 1 for the vaccination) and they may delay 1 or both visits until they have another reason to consult their GP. This possibility will be investigated in analyses of VC at 6 and 12 months, as will whether adolescents vaccinated at school receive the second dose of the vaccine.

## Conclusions

In February 2023, after the end of the PrevHPV trial, the French government announced that both doses of the HPV vaccine would be offered free of charge to all grade 7 pupils, at school, from autumn 2023.^50^ Preliminary reports show that approximately 10% of pupils have received the first dose, with much concern raised around unfounded vaccine safety concerns. The results of the PrevHPV trial, and the evaluation of its implementation in particular, could provide vital information on how to best carry out this policy and improve VC.

## Notes

Supplement 1.
Trial Protocol

Supplement 2. **eTable.** Characteristics of the 61 Municipalities Meant to Implement a School-Based Component, Depending on Whether Its School Had Dropped Out

Supplement 3.
Nonauthor Collaborators

Supplement 4.
Data Sharing Statement
