# Supplementary material for: Effectiveness of a School- and Primary Care–Based HPV Vaccination Intervention: The PrevHPV Cluster Randomized Trial
Source: JAMA Netw Open. 2024 May 23;7(5):e2411938. doi: 10.1001/jamanetworkopen.2024.11938 (PMC11117086; doi:10.1001/jamanetworkopen.2024.11938)
Supplement: Supplement 1. — Trial Protocol [file jamanetwopen-e2411938-s001.pdf]

Reserved sponsor	Inserm number	IDRCB No.	CPP No.	CNIL or MR number	No. Clinical Trial
	C19-54	2020-A02031-38	<…>	<…>	<…>
	Regulatory Qualification	☐ RIPH of 1° drug<br>RIPH of 1° innovative therapy drug<br>☐ RIPH of 1° medical device<br>☐ RIPH of 1° excluding health products		☒ RIPH of 2° with minimal risks and constraints<br>☐ RIPH of 3° non-interventional	

Evaluation of a multi-component intervention aimed at improving the acceptability of vaccination against Human PapillomaVirus (HPV) in France

“PrevHPV” trial – Experimental phase

VERSION N°1.0 - 07/10/2020

CONFIDENTIAL

☐ **Monocentric** ☒ **National**

☒ **Multicenter** ☐ **European/International**

Sponsor:
Inserm - Clinical Research Center (PRC)
Biopark, Building A, 8 rue de la Croix Jarry,
75013 Paris

Contact:
Layide ROUFAI
layide.roufai@inserm.fr
rqrc.siege@inserm.fr
Phone: 01 48 07 34 15/Fax: 01 44 23 67 10

1. RESEARCH RATIONALE – WORKING HYPOTHESES ... 4
2. OBJECTIVES ... 13
2.1. Primary objective ... 13
2.2. Secondary objectives ... 13
3. OUTCOMES ... 13
3.1. Primary outcome ... 13
3.2. Secondary outcomes ... 14
4. STUDY DESIGN ... 19
4.1. Type of research ... 19
4.2. Research Method ... 19
4.3. Provisional research timetable ... 21
5. SELECTION OF THE POPULATION ... 23
5.1. Populations under study ... 23
5.2. Inclusion and non-inclusion criteria ... 23
5.3. Selection methods ... 24
6. DISTINCTION BETWEEN USUAL CARE AND RESEARCH ... 26
7. IMPLEMENTATION OF THE STUDY ... 26
7.1. Selection ... 26
7.2. Inclusion, information, and participation agreement ... 26
7.3. Request for exemption from the collection of consent ... 27
7.4. Evaluation of the effect on knowledge, attitudes, beliefs and practices ... 28
7.5. Follow up ... 29
7.6. Expected duration of participation ... 29
7.7. Stopping one's participation in the research ... 29
7.8. End of research ... 30
8. INTERVENTIONS EVALUATED IN THIS PROTOCOL ... 30
9. SAFETY ... 38
9.1. Definitions ... 38
9.2. Responsibilities of the investigator ... 38
9.3. Potential risks of research and what to do in the event of an adverse effect ... 38
10. DATA COLLECTION AND PROCESSING ... 39
10.1. Description of collected data ... 39
10.2. Source data and documents ... 41
10.3. Data circuit ... 42
10.4. Conservation of research documents ... 42
11. STATISTICAL ANALYSIS ... 43
11.1. Responsible for statistical analysis ... 43
11.2. Sample size calculation ... 44
11.3. Description of the statistical analysis plan ... 44
12. CONFIDENTIALITY ... 47
12.1. Terms of confidentiality with respect to individuals ... 47
12.2. Terms of confidentiality with respect to the research ... 47
13. COMMUNICATION ... 48
13.1. Conditions relating to the publication of results ... 48
13.2. Modalities relating to the summary of the final report and the final report ... 48
13.3. Methods for informing people participating in research about its overall results ... 48
13.4. Conditions relating to press communication ... 49

14. PROTECTION OF INDIVIDUALS 49
14.1. Ethical justification of the protocol 49
14.2. Suitability of location for research 49
14.3. Ethical and regulatory provisions 50
14.4. Committee for the Protection of Persons (CPP) 50
14.5. ANSM 50
14.6. CNIL 50
14.7. Insurance and financing 51
15. GOVERNANCE AND COMMITTEE 51
15.1. Steering Committee (SC) 51
15.2. Monitoring committee 52
16. QUALITY ASSURANCE 52
16.1. Description 52
16.2. Monitoring (research quality control) 52
17. SUBSTANTIAL CHANGES TO THE PROTOCOL 52
18. REFERENCES 63

1. RESEARCH RATIONALE – WORKING HYPOTHESES

Human papillomavirus (HPV) infection is the most common sexually transmitted infection worldwide. More than 200 genotypes of the virus exist. Persistent infection with certain genotypes is associated with an increased risk of cancer (1). Several anatomical sites can be affected: cervix, vagina, vulva, penis, anus and oropharynx.

Cervical cancer is the fourth most common cancer in women in terms of incidence, with 570,000 new cases and 311,000 deaths in 2018 worldwide (2). In France, it is estimated that the number of new cases was nearly 3,000 in 2018, for 1,117 deaths (3). However, cervical cancer is one of the cancers that could be largely prevented, thanks to vaccination (4). Vaccination is effective for 90% of oncogenic HPV virus infections and the latest data show a reduction in the incidence of cancers of the cervix, anal canal and ENT sphere (5). Furthermore, the Gardasil 9® vaccine also provides protection against anogenital warts (6, 7). Since 2018, WHO has called for the implementation of actions to accelerate the eradication of cervical cancer worldwide (8). In France, since 2007, the High Council for Public Health has recommended vaccinating young girls from the age of 14 (9), before exposure to HPV infection, with a catch-up possible for adolescents and young women aged 15 to 23 who have not had sexual intercourse or at the latest the year following the start of their sexual life. Standard vaccination corresponded to 3 injections (0, 2, 6 months for the quadrivalent vaccine and 0, 1, 6 months for the bivalent vaccine) until 2014. Currently, anti-HPV vaccination is recommended for all young girls from 11 to 14 years (10,11) according to a 2-injection schedule, with a possible catch-up for 15-19 year olds who have not initiated vaccination. However, several recent works suggest a comparable efficiency of 1. 2 or 3 doses and clinical trials are underway to evaluate the effectiveness and durability of vaccination limited to a single injection (12). Vaccination of immunocompromised people aged 9 and over as well as men who have sex with other men is also recommended (13). Finally, following the launch of a public consultation dated 10/30/2019, the High Authority for Health (HAS) has recommended since 12/16/2019 the extension of vaccination to boys aged 11 to 14 (universal vaccination) with their planned integration into the vaccination schedule in 2020 (14), and reimbursement by the Statutory Health Insurance from January 2021.

The HPV vaccination coverage (VC) in France is one of the lowest in Europe; it is 29.4% for one dose in girls aged 15 and 23.7% for the complete regimen at 16 years (15), which is well below the objective of 60% set by the National Cancer Control Plan 2014-2019 (16,17), but also below the VC observed in most other European countries (18-20). Beyond the low VC, these figures also show that the main problem in France is the initiation of vaccination since 80% of adolescents who initiate vaccination complete it (injection of a 2nd dose). Furthermore, VC at the age of 15 went from 31% for girls born between 1994 and 1996 to 20% for those born in 2000.

This decline is likely the consequence of controversies over the HPV vaccine, its safety having been questioned, and in particular the potential risk of occurrence of autoimmune diseases following vaccination. Available in Europe in 38 countries for more than 10 years, with a total of more than 270 million doses injected until 2017, the data to date seems sufficient for the GACVS (Global Advisory Committee on Vaccine Safety, WHO) to consider the HPV vaccine as “extremely safe” in its July 2017 report (23). The risk of anaphylaxis has been characterized as approximately 1.7 cases per million doses. Pharmacovigilance has not attributed any serious adverse events to this vaccine (24-27), and in particular no autoimmune disease, except for a weak possible association with Guillain-Barré syndrome reported in a study conducted in France (28). However, this association was not confirmed by other subsequent studies (29,30), including a large one carried out in the United Kingdom among a population to whom 10.4 million doses had been administered. In this context of controversy, the medical community debated the benefits and risks of the HPV vaccine and a petition was sent to the Ministry of Health in May 2014 calling for a parliamentary inquiry into this subject (31). In reaction, in 2017, the GACVS expressed concerns about the regular dissemination of misleading case reports and unsubstantiated claims contradicting safety studies covering several million people and comparing risks for a wide range of endpoints in vaccinated and unvaccinated subjects. They stressed the importance of providing vaccine policy makers and other stakeholders with clear and accurate summaries of vaccine safety information, to help them make evidence-based decisions.

The optimization of anti-HPV vaccination in France has been the subject of various measures by successive governments and health authorities since 2009. Measure 13 of the 2009-2013 Cancer Plan aimed to increase the VC of 14-year-old girls through the dissemination of public and appropriate information to the target population and parents (32). The 2014-2019 Cancer Plan focused on free access to HPV vaccine via vaccination centers and on the mobilization and training of health professionals. In 2017, the HAS declared cervical cancer prevention through vaccination and pap smear screening to be a public health priority (33). This statement is the consequence of the low VC, hundreds of deaths and thousands of infections in the years to come being avoidable with vaccination (34),

However, actions aimed at improving HPV VC in France have had little effect and do not seem to meet existing needs. They are implemented by different stakeholders and are not coordinated. The national level mainly acts on recommendations and communication with the various stakeholders (regional health agencies, health professionals, general public) and finances vaccination centers certified by regional health authorities. The region mainly intervenes on access and communication. The department acts on access (financing and vaccination of the public), communication and monitoring of the vaccinated population.

In the context of vaccination in general, GPs (GPs) play a vital role, but to date remain poorly targeted in actions aimed at improving VC. Daily, these health professionals are confronted with the phenomenon of vaccine hesitancy (35) and due to their proximity to young people and their parents, they are a significant source of information and can thus influence them in their decision-making in favor of adherence to anti-HPV vaccination (36).

1.1. Summary of results of non-clinical trials and clinical trials available and relevant to the research in question

- Vaccine hesitancy

The phenomenon known as vaccine hesitancy may partly explain the low VC observed in France (37,38). It was defined by the WHO as the delay in accepting or refusing vaccination despite the availability of vaccination services. Influenced by many factors, it results in a delay in vaccination, incomplete vaccination (only one dose) or non-vaccination.

In an international study conducted by the 'Vaccine Confidence Project' group, France was identified as the European country where the population had the least confidence in the safety of vaccines (39). The parental decision to vaccinate their child or not is a complex social process influenced by multiple factors such as information received or sought from different sources (doctors, peers, social media, etc.), experiences, emotions, social norms. and environmental factors (40). The place of health professionals in vaccination decision-making is also important, according to the results of several studies conducted in France (41,42). In a national panel of 1,712 French GPs, 28% of them did not recommend HPV vaccination to target adolescents or their parents (43). A systematic review of the literature on the determinants of HPV vaccine hesitancy in Europe (44) showed that the main concerns were related to insufficient and inadequate information about vaccination, potential adverse effects of the vaccine, trust towards health authorities and the perceived low effectiveness of the vaccine.

As part of the REMPAR program (45-48), one of the teams involved in the project PrevHPV worked on the representations and practices of HPV vaccination in France. The results showed overall good acceptability of vaccination among GPs; those hesitant to vaccinate have difficulty broaching the subject with their patients (nature of vaccination (STI prevention), fear of parents' reaction). Mothers hesitant to vaccinate their daughters fear adverse effects and expect a lot from doctors to reassure them. Adolescent girls and their mothers play a major role in vaccination decision-making. Socially advantaged adolescents were more frequently vaccinated and demonstrated more developed prevention behaviors.

- Levers and barriers to vaccination

A systematic review of the literature on improving HPV VC made it possible to identify the following levers: doctor's recommendation, parental acceptance, peer motivation and coverage by statutory health insurance (49). The barriers identified relate to the cost of the vaccine, parental concerns (non-sexually active child, safety of the vaccine, belief that the vaccine would encourage sexual activity), lack of information and the preference to wait until their child is older.

A meta-analysis showed that doctor's recommendation of vaccination was the major factor influencing parents' decision to vaccinate their child, followed by concerns about vaccine safety. Partial coverage of the vaccine by statutory health insurance and out-of-pocket costs have been identified as factors weakly associated with the parental decision (50). Ethnic, cultural, and educational factors may also play an important role in VC (51).

Specifically for France, fewer data is available, but certain factors stand out as barriers to HPV vaccination: lack of knowledge about the infection and associated diseases and the absence of a proposal by a health professional (52,53), as well as the perception of an ineffective vaccine with a risk of serious side effects (54). The impact of socioeconomic status varies, but we note that a disadvantaged environment or poor access to medical consultation can be a barrier to vaccination (53,54). In the 2016 Health Barometer, around 54% of women aged 15 to 25 and parents of girls aged 11 to 19 considered ("completely" or "somewhat") that the vaccine could cause serious side effects (54).

- Vaccine safety

In a systematic review of the literature on the perceived risks of vaccines among the European population, the most common concerns were about unknown side effects that may develop long after vaccination. Many studies have reported concerns about the lack of clear information about HPV infection and the vaccine, as well as parents' feeling that they do not have the knowledge to make informed decisions (55). A systematic search of web activity using Google Trends shows that the HPV vaccine is a popular topic and that users are searching for information, including possible risks (56).

- Analysis of HPV vaccination preferences

Conjoint analyzes or discrete choice experiments are increasingly used to explore user preferences for healthcare interventions, particularly preventive interventions, from a social marketing perspective. Concerning HPV vaccination, previous joint analyzes have focused on the characteristics of the vaccine (effectiveness, price) and did not include communication content on the formulation of the information on the safety profile of the vaccine, social conformity or the potential for indirect protection. For example, a previous conjoint analysis in French students suggested that the vaccine's safety profile and controversy about it dominated theoretical acceptance of the vaccine (57). If this is confirmed for HPV vaccine in current target populations, interventions will need to address this issue. However, the explicit denial of rumors does not seem to have a positive impact (58). Joint analysis of preferences for vaccination programs can help narrow the gap between studies on the determinants of vaccination status or qualitative work and the design of intervention strategies (59). To progress in the design of interventions, it is necessary to understand under what conditions people who do not accept vaccination might change their attitude and request vaccination. To better understand how vaccination decisions are made in different population groups, these techniques also allow pre-testing individual interventions, particularly the content of communication, incentives or recommendations, in hypothetical scenarios.

- Comparison of vaccination policies in Europe

A systematic analysis of the literature associated with a cross-sectional survey of the European Center for Disease Prevention and Control (ECDC) in 31 European countries, carried out by one of the teams involved in the PrevHPV project (20), showed that countries where HPV VC was high (10 countries where VC is > 70%) have implemented (i) a structured systematic vaccination program including invitation to be vaccinated and an organized system of postal or telephone reminders, (ii) vaccination of target populations in schools, (iii) coverage of the cost of vaccination.

- <u>Evaluation of interventions on knowledge, attitudes and practices regarding infectionHPV and its vaccination</u>

According to the transtheoretical model developed by Prochaska and DiClemente (60), vaccination intention, which is decisive in the process of “vaccine decision” or “vaccine acceptance”, can be located between the phases of (pre)contemplation one side, and planning and action on the other. Unlike vaccination status, which is strongly influenced by the “access to vaccination” factor, vaccination intention is not dependent on access, although it is dependent on the perception of accessibility of vaccination. It is also conditioned by a vaccination offer: either by a doctor for an immediate prescription, or by communication which encourages people to seek access to vaccination.

In the literature, different concepts exist to explain or predict vaccination intention. The concepts of knowledge, attitudes, beliefs, and practices (KABP) have been used in prevention research, either to describe gaps in communication about a disease or a preventive practice in a given population, or to evaluate the impact of interventions which aim to improve communication. Thus, Grandahl, Liu then Paskett notably used KABP-type questionnaires to evaluate the effect of interventions on HPV in students and parents (61-63).

The Health Belief Model (HBM) has provided a theoretical framework for the development of vaccination confidence scales, such as the Vaccine Confidence Scale (VCS) developed by Gilkey (64). For HPV vaccine, the Carolina HPV Immunization Attitudes Scale (CHIAS) was developed by McRee et al. to provide interventional research on HPV with a standardized and validated tool (65). However, this tool is only validated among parents of young North American women. Gilkey et al. then developed a scale adapting the VCS to HPV, taking into account CHIAS. Richman et al. also proposed a version close to the CHIAS, tested cognitively, also in the United States (66). Finally, Perez et al. validated a questionnaire destined to parents of boys in Canada, in English and in French (67).

More recently, the notion of vaccine hesitancy has provided a deeper understanding of the antecedents of vaccination intention. First, MacDonald et al. presented the "3C model", which identifies "Complacency" (denial of the usefulness of the vaccine), lack of "Confidence" (in the safety of vaccines and the system of surveillance) and a perception of low “Convenience” (ease of access) as factors in vaccine hesitancy, working against vaccination intention (68). Betsch et al. expanded this framework to 5C, adding 2 additional dimensions, namely “Calculation” (of individual necessity given the vaccination of others) and “Collectivity” (motivation by a collective goal) (69). Finally, the ConjointVac studies (70) have illustrated the importance of “Social conformity” (the descriptive social norm) in the theoretical vaccination decision, which can therefore be considered as the sixth antecedent of vaccination intention. So we are talking about the “6C model”.

The two conceptual frameworks “KABP” and “6C” are not in contradiction, but can complement each other in the form of a matrix, particularly for the “knowledge” and “attitudes” dimensions. For example, confidence in vaccination can be analyzed in the form of knowledge, for example about the pharmacovigilance system and studies suggesting the safety of a vaccine, or in the form of attitude, representing the absence of concern or of unjustified fear. A practice may be to seek information about the safety of a vaccine from a reliable source or to tell other people that the vaccine is safe. In this perspective, vaccination intention and administration are the attitude and practice that arise from these antecedents.

- <u>Evaluation of interventions aimed at increasing HPV vaccination coverage</u>

There are currently few studies evaluating interventions aimed at improving HPV vaccination coverage, and none have been carried out in France. Two systematic reviews of the literature of this type of intervention in high-income countries have been carried out by Walling (71) and Vollrath (72), respectively, and draw largely on US studies. They are summarized below. The interventions described in the literature had as targets (alone or in combination) adolescents, parents and health professionals.

Interventions targeting adolescents

One study evaluated the impact of a massive information campaign in the United States targeting adolescent boys (73), which resulted in a nonsignificant increase in the HPV vaccination rate. Two studies evaluated behavioral interventions in schools (message framing) but did not find a significant improvement in vaccination rates (74,75). Two studies evaluated access to free vaccination through school: in the first, adolescents living in areas with a low VC were invited to a health center to be vaccinated, which increased local vaccination coverage from 5% to 59% (the national average) (76). The second study encountered many implementation difficulties in providing access to HPV vaccination clinics but found that initiation of vaccination was higher in schools where education campaigns were implemented compared to those where no education was offered (6% vs. 1%) (77). The majority of other available studies evaluated reminder messages to parents and adolescents, without making any difference between these two target populations, or interventions combining adolescents, parents and professionals (presented below). They suggest a small but significant effect on HPV VC.

Interventions targeting parents

Only one study evaluated an intervention offering information to parents. It consisted of 10-minute education sessions for low-income parents in the United States and showed a 16% increase in vaccination (78). The majority of other studies evaluated reminder messages to parents and adolescents, and as noted above did not differentiate between these two populations. Although one study reported no effect on vaccination (possibly due to inadequate follow-up duration) (79), most studies found significant improvements in complete vaccination rates over time between 10% and 25% by using SMS, email or telephone reminders (80-85).

Interventions targeting health professionals

Ensuring that health professionals recommend the HPV vaccine more to their patients is one of the key levers for improving VC (86-87). Better arming professionals to talk about HPV vaccination by providing them with knowledge, better self-confidence and societal and collegial support (88-89) should therefore appear in the usual vaccination protocols as well as in the tools put in place to address the usual barriers to vaccination (90).

Interventions aimed at educating professionals or working with reminders without combining the two have not shown a uniform effect across studies. More recently, a study including five methods of intervention with health professionals (information leaflet, website for parents, images of the disease, decision support and communication training) showed an increase in vaccination of 10% for initiation

(+4% for complete vaccination) (91). Generally, studies have shown that interventions targeting healthcare professionals are more effective when they combine reminders and education (92). A support tool for doctors (AFIX) developed by the American Center for Disease Control (CDC) including evaluation, feedback, incentives and exchange with peers also showed low but significant effectiveness on the anti-HPV vaccination rate (93, 94).

Interventions targeting adolescents, parents and health professionals

Significant effects have been observed by combining interventions aimed at parents/adolescents and health professionals. A study of an intervention combining reminders to parents and reminders and education of professionals showed an absolute increase of 9% in the intention to vaccinate and 13% in the complete vaccination rate. This combined effect of the different components was greater than that observed with each component evaluated separately (92). Similarly, a US study combining patient education and telephone reminders with physician alerts and a script to address patient concerns showed a 9-fold increase in full vaccination rates (80).

Potential role of e-health in increasing VC

A systematic review of communication tools aimed at improving the initiation and completion of HPV vaccination (such as reminders, SMS, emails, interactive videos on computer, etc.) supports the use of this type of communication tools (95). In addition, a review of different systematic reviews resulted in a recommendation for the use and evaluation of e-health technologies (defined by the WHO as “the secure and economically advantageous use of information technologies and communication in support of health and health areas") to encourage and increase adherence to vaccination (96).

- Avenues for improving the acceptability of HPV vaccination in France

Analysis of the literature on HPV vaccination highlights the following points:

(i) A lack of knowledge about the infection and associated diseases (52,53), as well as the perception of an ineffective vaccine with a risk of serious side effects (54) is associated with a low vaccination rate. In a review of the literature on adolescent vaccination in general, Dempsey identified among promising interventions the use of educational materials and websites to train/inform parents (97). In another study evaluating the effect of a multi-component intervention to improve anti-HPV vaccination coverage, the authors showed that this coverage was better in schools that had implemented an adolescent education campaign (98). However, no study of this type has been conducted in France.

(ii) Health professionals, and GPs in particular, play a fundamental role in the decision of adolescents and their parents to proceed with vaccination. Lemaitre et al. showed that an educational information session on immunization based on motivational interviewing techniques, put in practice during mothers' postpartum hospitalization could improve immunization coverage during infancy (99). These authors described the usefulness of motivational interviewing. Furthermore, a training program on communication skills for French GPs has already been successfully tested to improve colorectal cancer screening (100). Decision aids are interventions that support patients by making their decisions explicit, providing information about options and associated advantages/disadvantages, and helping to clarify the congruence between decisions and personal values (101). In the Cochrane meta-analysis on decision aids (which does not only focus on vaccines), the median effect of a decision aid on the duration of the

medical consultation was 2.6 minutes (102). In the field of vaccination, few decision support tools have been evaluated.

(iii) Compared to European countries where vaccination coverage is high, access to anti-HPV vaccination in France is not facilitated, on two levels: (1) at the financial level (high cost of the vaccine, 35% of the cost not covered by health insurance), and (2) at the organizational level (pathway of access to vaccination with medical prescription, dispensation by the pharmacy and return to the doctor for administration). The majority of European countries where vaccination coverage is high have introduced systematic vaccination in schools (20).

In this context, a call for expressions of interest launched in June 2018 at the initiative of the Public Health Research Institute (IReSP) and the ITMO Public Health (AVIESAN) made it possible to identify eight teams distributed across the French territory to build and implement a research project in human and social sciences and epidemiology in relation to the acceptability of HPV vaccination among young girls of school age. A consortium made up of the eight selected research teams was formed (see full list of teams in “15. Governance and Committee”). This consortium constitutes the steering committee (SC) of the project and meets monthly in order to develop, implement, monitor the progress of the project, carry out statistical analyzes and valorization of the project (report, publications, conference communications). Each SC team is represented by a scientific manager (see part 15), the scientific manager of Team 1 also being the coordinating investigator of the research. To implement and monitor the progress of the project, the following were specifically recruited: a project/team manager (for all teams except 6 and 8, exclusively in charge of analyses), and a study coordinator assigned to Team 1 who is responsible for coordinating all the teams and phases of the project. Between September 2018 and February 2019, the SC constructed and wrote a research project and a funding request, which were submitted to an independent evaluation committee made up of European public health experts and representatives of national authorities (Inserm, Public Health France, DGS, INCa, ITMO Public Health, ITMO Cancer, DGESCO, ARS Ile de France). At the end of April 2019, this evaluation committee validated a second version of the project and granted the funding requested as part of the 2014-2019 Cancer Plan.

The objective of the project selected by the evaluation committee is to identify, co-construct, implement and evaluate a multi-component intervention aimed at improving the acceptability of HPV vaccination by the target populations and, *in fine*, improve VC. This project is divided into three phases:

(i) A diagnostic phase (01/10/19 – 30/06/20) aimed at identifying knowledge, beliefs, attitudes, preferences, or levers and barriers to HPV vaccination among adolescents in middle school, their parents, middle school professionals, and GPs. Data on knowledge, beliefs, attitudes as well as the barriers and levers to HPV vaccination in France do exist, but they are now old since they relate to the period preceding the modification of the target age and the vaccination schedule. In addition, they concern young girls, their parents and their GP but none concern National Education professionals or young boys. Furthermore, no study has to date explored users' preferences regarding HPV vaccination. This diagnostic phase is considered to be outside the Jardé law (human and social sciences research), and therefore does not require the approval of a Committee for the Protection of Persons (CPP). It was nevertheless submitted to the Inserm Ethical Evaluation Committee (CEEI), which issued a favorable opinion on 10/12/2019. The

various surveys (quantitative, qualitative, and joint analyses) carried out as part of this phase are currently being carried out, with data exploitation planned for the last quarter of 2020.

(ii) A co-construction phase of the multi-component intervention (01/10/19 – 30/11/20) with the various stakeholders. The intervention in question is broken down into the following three components:

1. An 'Education, Motivation, Mobilization' component targeting adolescents and their parents and using, among others, as human resources health students who have been trained in prevention within the framework of the Health Service (SeSa),
2. A 'Training of GPs' component with training in motivational interviewing and the provision of a decision-making tool previously developed by the SC,
3. A 'Facilitation of access to vaccination' component which proposes to initiate HPV vaccination in middle schools, via vaccination centers.

The content of each component was broadly defined by the SC based on the analysis of the literature and the French context, as part of the initial research project submitted to the evaluation committee in April 2019. Between 01/10/2019 and 31/12/2020, the exact content and precise organization (who does what? when? where? how?) of each component were defined by three working groups (one per component) integrating the different stakeholders (researchers from the operational committee, adolescents, parents, national education professionals, SeSa students, GPs, vaccination centers). Between 11/01/2020 and 12/30/2020, the content of each component will be adjusted/finalized based on the results of the diagnostic phase.

2. OBJECTIVES

2.1. Primary objective

The main objective of the research conducted as part of the experimental phase (PrevHPV trial) is to evaluate the effectiveness of three components of the intervention, each component being delivered either alone or associated with one or two others, on the vaccination coverage for adolescents (girls and boys) aged 11-14.

2.2. Secondary objectives

1. Evaluate the effect of the different components of the intervention, alone or combined, on (i) the vaccination intention of adolescents, parents and GPs, and (ii) knowledge, attitudes, beliefs and practices on infection and HPV vaccination of adolescents, parents, and GPs (including history of vaccine hesitancy), using the KABP-6C questionnaire.
2. Evaluate the impact of the socio-economic level of parents on the effectiveness of the different components of the intervention, alone or combined, on VC in adolescents aged 11-14 years.
3. Evaluate the efficiency (cost-effectiveness) of the different components of the intervention, alone or combined, among adolescents aged 11-14 years.
4. Evaluate the budgetary impact of the different components of the intervention, alone or combined. Note that this objective will only be carried out if the intervention under evaluation is cost-effective (secondary objective 3).
5. Evaluate the implementation of the different components of the intervention, and identify the levers and barriers to implementation at the individual and collective level to assess the transferability of the intervention to other contexts.

3. OUTCOMES

3.1. Primary outcome

To the primary objective correspond 1 primary outcome and 5 secondary outcomes (see first 2 lines of the table page 15) depending on the number of doses of vaccine administered (1 or 2 doses) and the time of measurement (2 months, 6 months or 12 months after the end of the intervention).

The primary outcome to assess the effectiveness of the different components of the intervention is the vaccination coverage rate (= prevalence of vaccination, i.e. at least 1 dose received) among adolescents aged 11-14 years, 2 months after the end of the intervention. This prevalence will be calculated overall, then separately for girls and boys.

Prevalence is defined as the number of adolescents aged 11 to 14 years who have received at least 1 dose of HPV vaccine (Cervarix®, Gardasil®, Gardasil9®) / the total number of adolescents aged 11 to 14 years. Prevalence before intervention implementation will be calculated among adolescents using the databases of the National Health Data System (SNDS, i.e. the national reimbursement database) (103), data from vaccination centers and family planning centers. We are aware that health insurance coverage for vaccination in boys starting in January 2021, pharmaceutical dispensation among

boys in the SNDS will most likely be close to 0. In the SNDS bases, we will count 14-year-olds who have received at least 1 dose in the last 4 years, 13-year-olds in the last 3 years, 12-year-olds in the last 2 years, and 11-year-olds in the past year.

The prevalence 2 months after the end of the intervention will be calculated using data from the SNDS, data from vaccination centers and family planning centers, and data from vaccination in middle schools. For the same dose of vaccine, it is therefore not possible for an individual to be present in several different data sources, and it is therefore not possible to count people twice. Indeed, if an adolescent (and their parents) decides to be vaccinated following the intervention, he or she has 4 possibilities of access to vaccination: be vaccinated by his or her attending physician (information available in the SNDS via the dispensation of the vaccine in a community pharmacy), get vaccinated in a vaccination center, get vaccinated in a family planning center, or get vaccinated at school as part of the PrevHPV intervention.

The 11-14 years old age group was chosen to calculate the prevalence of vaccination (despite the proposal to vaccinate 11-19 year olds in middle schools - see facilitation of access) because (i) 11-14 year olds are the target for initial vaccination (15-19 year olds = vaccination of catch-up), (ii) those over 14 are in the minority in middle schools.

As indicated above, the prevalence before and at 2 months will be calculated for the entire population of the age group, then separately for girls and boys.

3.2. Secondary outcomes

The secondary outcomes are of two types: (i) those common to the three components of the intervention, evaluated alone or combined; (ii) those specific to one of the three components.

3.2.1. Secondary outcomes corresponding to the objectives common to the 3 components

Specific objectives	Result	Outcome measure	Time of measure
Evaluate the effectiveness of the intervention on the prevalence of vaccination (at least 1 dose received), 6 and 12 months after the end of the intervention (Primary Obj)	Variation in the prevalence of vaccination (at least 1 dose) between before the start of the intervention and after the end (6 months and 12 months) among young people aged 11 to 14 years	Pre-intervention prevalence: number of young people aged 11 to 14 who have received at least one dose of vaccine (SNDS data, vaccination centers, planning centers) / the total number of young people in this age group.<br>Prevalence after the intervention: number of young people aged 11 to 14 who had received at least one dose of vaccine before the intervention + after the intervention (SNDS data + vaccination centers + planning centers + number of vaccines administered within middle schools) / the total number of young people in this age group.<br>Calculated globally and separately for boys and girls	Before the intervention<br>+ At 6 months and 12 months after the end of the intervention
Evaluate the effectiveness of the intervention on the prevalence of complete vaccination (2 doses received), 2, 6 and 12 months after the end of the intervention (Primary Obj)	Variation in the prevalence of complete vaccination (2 doses received) between before the start of the intervention and after the end (2 months, 6 months and 12 months) among young people aged 11 to 15 years	Prevalence before the intervention: number of young people aged 11 to 14 who received two doses of vaccine (SNDS data, vaccination centers, planning centers) / total number of young people in this age group.<br>Prevalence after the intervention: number of young people aged 11 to 14 who had received two doses of vaccine before the intervention + after the intervention (SNDS data + vaccination centers + planning centers + number of vaccines administered within middle schools) / the total number of young people in this age group.<br>Calculated globally and separately for boys and girls	Before the intervention<br>+ At 2 months, 6 months and 12 months after the end of the intervention
Evaluate the effect of the intervention on parents' intention to vaccinate their child (Secondary Obj 1)	Change in the percentage of parents declaring their intention to vaccinate their child between before the start of the intervention and after the end	KABP-6C questionnaire (see appendix 2)	Before the intervention<br>+ 2 months after the end of the intervention
Evaluate the effect of the intervention on whether parents report HPV vaccination in their child (Secondary Obj 1)	Change in the percentage of parents reporting having had their child vaccinated against HPV between before the start of	KABP-6C questionnaire (see appendix 2)	Before the intervention<br>+ 2 months after the end of the intervention

	the intervention and after the end		
Evaluate the effect of the intervention on the knowledge, attitudes and practices (including history of vaccine hesitancy) of parents with regard to HPV infection and HPV vaccine (Secondary Obj 1)	Before-after change in percentages or medians of scores obtained on the Likert scales of the questionnaire addressed to parents	KABP-6C questionnaire (see appendix 2)	Before the intervention<br>+ 2 months after the end of the intervention
Evaluate the effect of the intervention on the intention of Grades Eight and Nine students to be vaccinated (Secondary Obj 1)	Change in the percentage of students declaring their intention to be vaccinated against HPV between before the start of the intervention and after the end	KABP-6C questionnaire (see appendix 2)	Before the intervention<br>+ 2 months after the end of the intervention
Evaluate the effect of the intervention on the fact that Grades Eight and Nine students declare anti-HPV vaccination (Secondary Obj 1)	Change in the percentage of students reporting having been vaccinated against HPV between before the start of the intervention and after the end	KABP-6C questionnaire (see appendix 2)	Before the intervention<br>+ 2 months after the end of the intervention
Evaluate the effect of the intervention on the knowledge, attitudes and practices (including history of vaccine hesitancy) of young people in Grades Eight and Nine with regard to HPV infection and HPV vaccine (Secondary Obj 1)	Before-after change in percentages or medians of the scores obtained on the Likert scales of the questionnaire addressed to Grades Eight and Nine students	KABP-6C questionnaire (see appendix 2)	Before the intervention<br>+ 2 months after the end of the intervention
Evaluate the effect of the intervention on the intention of GPs to offer vaccination to their patients (Secondary Obj 1)	Change in the percentage of GPs declaring that they offer vaccination to their patients between before start of the intervention and after the end	KABP-6C questionnaire (see appendix 2)	Before the intervention<br>+ 2 months after the end of the intervention
Evaluate the effect of the intervention on the knowledge, attitudes and practices (including history of vaccine hesitancy) of GPs with regard to HPV infection and the anti-HPV vaccine (Secondary Obj 1)	Before-after change in percentages or medians of scores obtained on the Likert scales of the questionnaire addressed to GPs	KABP-6C questionnaire (see appendix 2)	Before the intervention<br>+ 2 months after the end of the intervention
Evaluate the impact of socio-economic status on the prevalence of vaccination (1 dose, 2 doses), 2, 6 and 12 months after the end of the intervention (Secondary Obj 2)	Vaccination prevalence results (1 or 2 doses, and at each time of measure) calculated according to an ecological deprivation index (FDep)	Measure corresponding to each outcome<br>Economic status: SNDS data	Before the intervention<br>+ At 2 months, 6 months and 12 months after the end of the intervention

For secondary objective 3 (Evaluate the efficiency of the intervention), the outcome is the incremental cost-effectiveness ratio, expressed as additional cost to increase the vaccination coverage rate (= vaccination prevalence, i.e. at least 1 dose received) by 10% in adolescents aged 11-14 years, 2 months after the end of the intervention. The costs considered are the costs of the vaccine, the costs of the intervention and its implementation.

For secondary objective 4 (Evaluate the budgetary impact of the intervention), the outcome is the annual cost (financial and in terms of health gains) associated with the generalization of the national intervention in the short and medium term.

For secondary objective 5 (Evaluate the implementation of the intervention + levers and barriers to implementation), the outcomes are the following four criteria: (i) the dose of the intervention (dose of the intervention delivered and received by the target population), (ii) the fidelity of the intervention (gap between the planned intervention and the intervention actually implemented), (iii) the achievement of the target (participation of the target population in the different components of the intervention), and (iv) the adaptation of the different components of the intervention to different specific contexts (disadvantaged middle schools – REP / REP+, medical deserts, etc.).

3.2.2. Secondary outcomes corresponding to objectives specific to a component

Specific objectives	Result	Outcome measure	Time of measure
'Education, Mobilization, Motivation' component			
Evaluate vaccine hesitancy among health students and middle school staff (teachers / nurses / doctors / administrative staff)	Vaccine hesitancy	Vaccine Confidence Scale 5C Scale	Before the training of health students
Assess health students' knowledge of HPV infection and HPV vaccine	Percentage of students providing accurate answers to the questionnaire	Multiple choice quiz on HPV infections and prevention	Before the training of health students
Evaluate health students' satisfaction with the intervention (Secondary Obj. 5)	Student satisfaction	Self-administered questionnaire	After the intervention
Evaluate the satisfaction of middle school professionals with the intervention (Secondary Obj. 5)	Satisfaction of middle school staff	Self-administered questionnaire	After the intervention
Evaluate the use of the e-health tools by the target population (Secondary Obj. 5)	Use of tools in participating groups	Number of connexions	Monthly after the start of the intervention
Evaluate user satisfaction with e-health tools (Secondary Obj. 5)	User satisfaction	Self-administered questionnaire	After the intervention
'Training of GPs' component			
Evaluate the interest of GPs in training in motivational interviewing and the decision aid tool (Secondary Obj. 5)	Percentage of GPs participating in the training and using the decision aid tool	Number of healthcare professionals present and declaring use of the tool	During training sessions and after
Evaluate the effect of training on GPs' communication skills and decision support	Percentage of GPs declaring themselves competent in communication on the vaccine	Self-administered questionnaire	At 2 months, 6 months and 12 months after the intervention
'Facilitation of access to vaccination' component			
Evaluate acceptance of vaccination in middle schools (Secondary Obj. 5)	Percentage of young people aged 11 to 14 who initiated vaccination in middle schools	Number of doses of vaccine administered in middle school / number of young people eligible for vaccination within the school	Vaccination day
Evaluate the satisfaction of teenagers with regard to the intervention (Secondary Obj. 5)	Adolescent satisfaction	Self-administered questionnaire	After the intervention
Evaluate the satisfaction of middle school staff with the intervention (Secondary Obj. 5)	Satisfaction of middle school staff	Self-administered questionnaire	After the intervention

4. STUDY DESIGN

4.1. Type of research

The PrevHPV trial is a multicenter cluster randomized controlled trial with a 6-arm partial factorial design. The geographical areas covered by the trial correspond to the academies of Aix-Marseille, Clermont-Ferrand, Créteil, Grenoble, Lille, Limoges, Lyon, Nancy-Metz, Nantes, Reims, Strasbourg, Toulouse, Orléans-Tours, and Versailles. These academies were selected to have a diversity of profiles (geographic, behavior/vaccination) across mainland France, in collaboration with the General Directorate of School Education. Within the geographical areas covered by the trial, eligible municipalities are then identified (based on their characteristics and the participation of middle schools in the trial). The cluster, which is the randomization and evaluation unit, correspond to all the inhabitants in a municipality (identified by an administrative code, «code commune»).

The use of a factorial design makes it possible to evaluate the effect of each component of the intervention in isolation (compared to the absence of intervention and between each other), but also the effect of the association of the different components to identify whether it is additive, synergistic or antagonistic.

4.2. Research Method

A complete factorial design with 3 factors (= each of the 3 components) results in the constitution of 8 distinct groups. In our case, these 8 groups would be as follows:

- Group 1 benefiting from the 3 components 'Education, Motivation and Mobilization (EMM)' +'Training of GPs' + 'Facilitation of access to vaccination',
- Group 2 benefiting from 'EMM' + 'Training of GPs',
- Group 3 benefiting from 'EMM' + 'Facilitation of access to vaccination',
- Group 4 benefiting from 'Training of GPs' + 'Facilitation of access to vaccination',
- Group 5 benefiting from 'EMM' only,
- Group 6 benefiting from ‘Training of GPs' only,
- Group 7 benefiting from 'Facilitation of access to vaccination' only,
- Group 8 benefiting from none of the 3 components under evaluation (= control group).

However, not all of the above combinations are necessarily relevant. Indeed, it does not seem appropriate to offer vaccination on the school premises (facilitated access) to young people and their parents without them having first benefited from education on HPV infection and its vaccination (EMM), allowing them to make informed decisions. This is the reason why the 'Facilitation of access to vaccination' component will not be implemented without the 'EMM' component, and groups 4 and 7 will therefore not be formed.

Consequently, our partial factorial design will include the following 6 groups:

- ***Group 1*** benefiting from the 3 components 'Education, Motivation and Mobilization (EMM)' +'Training of GPs' + 'Facilitation of access to vaccination',

- ***Group 2*** benefiting from 'EMM' + 'Training of GPs',

- ***Group 3*** benefiting from 'EMM' + 'Facilitation of access to vaccination',

- ***Group 4*** benefiting from 'EMM' only,

- ***Group 5*** benefiting from 'Training of GPs' only,

- ***Group 6*** benefiting from none of the 3 components evaluated (= control group).

Each group is made up of 15 municipalities (see 11.2 Sample size calculation), making a total of 90 municipalities participating in the PrevHPV trial.

The 'Training of GPs' component of the 45 municipalities in groups 1, 2 and 5 is implemented from December 2020 to April 2021.

The 'EMM' component of the 60 municipalities in groups 1, 2, 3 and 4 is implemented from January to March 2021 and the 'Facilitation of access to vaccination' component of the 30 municipalities of groups 1 and 3 is implemented. work from February to April 2021.

A KABP-6C survey, at the individual level, repeated cross-sectionally (pre and post interventions) will make it possible to answer secondary objective 1 of the PrevHPV trial and thus to assess knowledge, attitudes, beliefs and practices. The KABP-6C questionnaire will be administered to volunteers in the following populations:

- Students, girls and boys, enrolled in Grades Eight and Nine in one of the public or private middle schools in the 90 municipalities of groups 1 to 6 before the implementation of the components in middle schools (December 2020) and after (May 2021).
- Parents, fathers and mothers of girls and/or boys enrolled in Grades Six through Nine in one of the public or private middle schools in the 90 municipalities of groups 1 to 6 before the implementation of the middle school components (December 2020) and after (May 2021).
- GPs practicing regular consultations with adolescents in the 60 municipalities of groups 1, 2, 5 and 6 before the implementation of the component (December 2020) and after (April 2021).

Costs and implementation indicators are collected prospectively by the referent of each middle school with the help of the PrevHPV project manager of the geographical sector concerned, as the different components of the intervention are implemented.

Randomization groups

	With training of GPs	Without training of GPs
**Education, motivation, mobilization**	Education, motivation, mobilization + Training of GPs	Education, motivation, mobilization only
**Education, motivation, mobilization + Facilitation of access**	Education, motivation, mobilization + Facilitated access + Training of GPs Complete intervention	Education, motivation, mobilization + Facilitated access
**No Education, motivation, mobilization + No Facilitation of access**	Training of GPs only	No intervention

4.3. Provisional research timetable

These dates are provided for purely indicative purposes and cannot be considered definitive.

**Tasks**	**Beginning**	**End**
Requests for regulatory authorizations	02/01/2020	15/10/2020
**1) GPs**	01/12/2020	04/30/2021
1.1) KABP-6C assessment before intervention	01/12/2020	12/31/2020
1.2) Training	01/01/2021	03/31/2021
1.3) KABP-6C evaluation after intervention	01/04/2021	04/30/2021
**2) TEENS, PARENTS and MIDDLE SCHOOL PROFESSIONALS**	01/12/2020	05/30/2021
2.1) KABP-6C assessment before intervention	01/12/2020	12/31/2020
2.2) EMM	01/01/2021	03/31/2021
2.3) HPV vaccination	02/01/2021	04/30/2021
2.4) KABP-6C evaluation after intervention	01/05/2021	05/31/2021
Analysis and valuation of results	01/06/2021	01/06/2022

Duration of implementation of the research intervention: 7 months (December 2020 – June 2021)

Total duration of the research: 2 years

The training of GPs (see 8. Interventions evaluated in the context of the research) is an online training (e-learning type), with an equivalent of 4 hours of lessons, that the participating GPs will be able to access in at any time between January and March 2021.

The education, motivation, mobilization (EMM) sessions for middle school students and their parents (see 8. Interventions evaluated as part of the research) will take place in the middle school, with the active participation of health students. This education will be spread over several half-days for Grades 8 & 9 middle school students and a 2-hour information session for parents and all middle school teenagers. The directly targeted middle school students were restricted to Grades 8 & 9 students (and not 11-14 year olds, i.e. all classes in the middle school) for the following 2 reasons: (i) for a question of feasibility practice of implementing the intervention; (ii) education on HPV infection requires discussing the mode of transmission through sexual contact, but sexuality is only discussed from Grade 8, and so Directorate for school education prefers that we do not communicate on this subject in younger pupils. This component of the intervention will take place between January and March 2021.

Vaccination on the middle school site (see 8. Interventions evaluated as part of the research) will take place over 0.5 to 2 days for each eligible middle school, depending on its size and the number of middle school students wishing to be vaccinated. It will take place 4 to 6 weeks after the EMM intervention, between February and April 2021. Each middle school student concerned will be mobilized for approximately 30 minutes (trip to the infirmary, checking the health record and information, injection of the vaccine, monitoring of the effects of the vaccine for about fifteen minutes).

For each target population (middle school students, parents, GPs), a KABP-6C questionnaire will be offered before and after the implementation of the different components of the intervention.

In total, for middle school students benefiting from the EMM component, the duration of participation in the intervention will be a few days, between January and March 2021. For middle school students benefiting from the EMM and Facilitation of access to vaccination component, the duration participation in the intervention will be a few days, between January and April 2021. For GPs, the duration of participation in the intervention will be a few hours, between January and March 2021. In addition to participation in the interventions, the time taken to complete the questionnaires (KABP-6C, satisfaction questionnaire) before and after intervention(s) must be taken into account for each of the three populations above, estimated at approximately 15 minutes each.

Middle school students, their parents and GPs participating in PrevHPV research will be able to participate simultaneously in other research. There is no exclusion period.

5. SELECTION OF THE POPULATION

5.1. Populations under study

- **The target populations of the intervention are:**

(i) GPs practicing in the municipalities randomized to groups 1, 2, 5,

(ii) Middle school students enrolled in middle schools located in municipalities randomized to groups 1, 2, 3 and 4,

(iii) Parents of middle school students attending middle schools located in municipalities randomized groups 1, 2, 3 and 4.

Groups / Target populations	Middle school students	Parents of middle school students	GPs
Group 1 - EMM + Facilitation of access + Training of GPs	X	X	X
Group 2 - EMM + Training of GPs	X	X	X
Group 3 - EMM + Facilitation of access	X	X	/
Group 4 – EMM	X	X	/
Group 5 - Training of GPs	/	/	X
Group 6 - No intervention	/	/	/

Populations targeted by the PrevHPV trial

The effect of the intervention will be evaluated on:

(i) Adolescents (girls and boys) aged 11 to 14 residing in the 90 municipalities participating in PrevHPV research (primanry and secondary objectives),

(ii) Parents of middle school students attending a middle school located in the 90 municipalities participating in PrevHPV research (secondary objective 2),

(iii) GPs practicing in the municipalities randomized to groups 1, 2, 5 and 6 (secondary objective 2).

- **Others affected by this research**

Furthermore, National Education professionals as well as stakeholders (for example health students) who will participate in the various actions will be questioned using satisfaction questionnaires.

5.2. Inclusion and non-inclusion criteria

Inclusion in PrevHPV research is carried out at 2 levels:

(i) the inclusion of participating municipalities,

(ii) the inclusion of people participating in the research.

- **Inclusion of participating municipalities:**

The inclusion criteria for municipalities are as follows:

- Municipalities of the academies Aix-Marseille, Clermont-Ferrand, Créteil, Grenoble, Lille, Limoges, Lyon, Nancy-Metz, Nantes, Reims, Strasbourg, Toulouse, Orléans-Tours, Versailles,
- Municipalities in which a total of 1 to 4 middle schools (public or private) are located,

- Municipalities drawn at random from the group of 409 municipalities to be requested to participate in the research,
- Municipalities where all middle schools agree to participate in research.

- **Inclusion of people participating in interventions:**

Inclusion criteria

✓ For the intervention component 'Training of GPs' are:

- GPs practicing in the municipalities randomized to groups 1, 2, 5,
- Agreeing to participate in training in motivational interviewing and the use of the decision-aid tool.

Will not be included: (i) GPs having exclusively a Particular Mode of Practice, (ii) whose practice within the municipality is planned to stop during the first half of 2021, (iii) GPs with no internet access.

✓ For the intervention component 'Education, Motivation, Mobilization', are:

- Adolescents (girls and boys), enrolled in Grades 8 & 9 classes in middle schools (public and private) located in the municipalities randomized to groups 1, 2, 3 and 4,
- Parents of adolescents (girls and boys), enrolled in Grades 6 to 9 classes in middle schools (public and private) located in the municipalities randomized to groups 1, 2, 3 and 4, who agree to participate in an information/education session,

✓ For the intervention component 'Facilitation of access to vaccination', are:

- Adolescents (girls and boys), enrolled in middle schools (public and private) located in municipalities randomized to groups 1 and 3, in Grades 6 to 9 classes,
- Aged 11 to 14 on the date of injection, with possible catch-up among 15-19 year olds for those who have repeated a grade, in accordance with the recommendations[1],
- Not vaccinated against HPV on the date of offer of vaccination in middle school (April – May 2021)[2],
- Whose parents (or representatives of parental authority) agree to the initiation of HPV vaccination in middle school,
- Not presenting a contraindication to HPV vaccination (hypersensitivity to the active substances or to one of the excipients of the vaccine, severe acute febrile illness on the date of injection)[3]
- Affiliated with a social security scheme or beneficiary of such a scheme[4].

5.3. Selection methods

Recruitment is carried out in two stages: (i) the selection of eligible municipalities, (ii) the selection of participating middle schools, making it possible to recruit the 90 municipalities, (iii) the randomization of the components of the intervention, and (iv) the identification of GPs to participate in the training.

1 Current vaccination recommendations (9-13) target girls and boys aged 11 to 14, with a catch-up possible from 15 to 19 years old. However, the vast majority of middle school students are between 11 and 14 years old. The exact age of middle school students on the planned date of the vaccine injection will be verified with the middle school administration. It is not possible to vaccinate middle school students under 11 years old, as this age is not indicated in the authorization for the vaccine.

2 The absence of HPV vaccination on the date of offer of vaccination in middle school will be verified with the parental authority at the time of the request for vaccination authorization, and checked against the health record at the time of injection.

3 The absence of contraindication to vaccination will be verified by the doctor of the vaccination center carrying out the injection on site at the time of the procedure, by questioning the student and checking the vaccination record.

4 Affiliation to a social security scheme will be verified with the middle school administration between the date of authorization from parental authority and the planned date of vaccination.

- **Selection of eligible municipalities:**

Information from academies (rectorates and diocesan management)

Each selected academy (rectorates and diocesan directorates) is informed in advance of the research by a note from the director of the DGESCO, and a letter detailing the experiment from the PrevHPV SC.
At the same time, 2049 municipalities in the selected academies meeting the inclusion criteria defined above were identified using data extracted from the data.education.gouv.fr website. These municipalities are then stratified according to the department and the FDep (ecological indicator of deprivation, dichotomized into 2 classes: > national average, < average). A random draw stratified on these two criteria made it possible to identify 409 potentially participating municipalities (appendix 3).

- **Selection of participating middle schools[5]:**

For each of the 409 municipalities selected, the principals and directors of each middle school located in the municipality are contacted by telephone, after having been informed by mail of PrevHPV research. This telephone contact allows us to provide additional information on the research and to request the middle school's agreement (or not) to participate. Ultimately, the participating municipalities are those for which all the middle schools located there (public + private) agree to participate in the research. The total number of municipalities to recruit is 90. If this number is not reached after contacting the 409 municipalities previously selected, a second stratified draw – using the same methods as the first – is carried out from the initial file of the 2049 (- 409) municipalities.
The principals and directors of each middle school are informed that, depending on the results of the randomization of the intervention components, participating in the research may involve benefiting from:

(i) EMM + facilitation of access to vaccination,
(ii) EMM alone,
(iii) neither (control group),

A commitment to respect the result of the randomization will be requested from the principals/directors of the participating middle schools, that is to say to facilitate the implementation of the EMM components and/or facilitation of access to vaccination if they are concerned.

- **Randomization of intervention components:**

For each of the 90 participating municipalities, a random draw stratified on the department and the deprivation group (according to the FDep) makes it possible to randomly assign one of the 6 research groups to each municipality. At the end of this draw, the DGESCO, the rectorates and the diocesan management, as well as the principals and directors of the middle schools are informed by postal mail from the PrevHPV's SC of the components the middle school will benefit from.

5 The list of municipalities cannot be established until the middle schools agreeing to participate are known. Due to the COVID-19 lockdown, organizational changes within middle schools following the end of lockdown, the upcoming summer holidays and potential changes of principals in middle schools at the next school year, it was not possible to establish the list that will participate in the project next year. As soon as it is established, this list will be communicated to the members of the CPP

In the same way, the mayors of the 90 participating municipalities are informed of the research and the allocation group of their municipality by post and the Departmental Councils will also receive a letter on this subject. They may also be contacted by telephone to discuss contextual elements that could facilitate the implementation of the intervention in their territory.

- **Identification of GPs to participate in the training:**

For each of the 45 municipalities in groups 1, 2 and 5, the list of GPs practicing within each of them is established. These GPs are then contacted by telephone, after having been contacted by mail, to verify their eligibility and offer them the opportunity to participate in motivational interview training and the decision-aid tool.

6. DISTINCTION BETWEEN USUAL CARE AND RESEARCH

This research does not modify the usual care of the targeted populations. Regarding interventions with adolescents, we offer tools that may or may not be used because the project is essentially based on what already exists. Indeed, human papillomavirus (HPV) can be addressed through different disciplines and through different themes such as those linked to vaccination or sexuality education provided during cycle 4 (Grades 7 to 9).
Through the intervention offering vaccination within middle schools, the project simply aims to facilitate adolescents' access to vaccination by putting them in direct contact with Mobile Vaccination Teams.
The only procedures added as part of this research are self-administered questionnaires (KABP-6C + satisfaction questionnaires).

7. IMPLEMENTATION OF THE STUDY

7.1. Selection

See '5.3 Selection methods'

7.2. Inclusion, information, and participation agreement

The selection process, information – participation agreement, and inclusion is as follows (see paragraph 5.3 Selection methods):

- Identification of eligible academies,
- Information from the rectorates of eligible academies and diocesan directorates of eligible departments by mail (DGESCO director letter + PrevHPV's SC letter),
- Identification of 409 eligible municipalities in eligible academies,
- Information by mail then telephone from the principals and directors of the middle schools of the 409 municipalities + request for participation agreement (→ identification of the 90 participating municipalities),
- Randomization of the allocation group (group 1 to 6) of the components for each participating municipality,
- Information for principals and directors of each middle school in their assigned group,
- Information for the mayors of each of the 90 participating municipalities and the corresponding

departmental councils,

- Information for GPs in municipalities in groups 1, 2 and 5, verification of eligibility and agreement to participate in training.

For each middle school of the 60 municipalities randomized to groups 1, 2, 3 and 4 (benefiting from EMM alone or EMM + facilitation of access to vaccination), the principal/director is in charge of informing the staff (health, teaching, administrative) of his middle school of the component they will receive. It will be essential that a referent be appointed within each middle school (school nurse/doctor, biology teacher, administrator, etc.) to be the main contact for the PrevHPV SC and to act as a link between the research teams and the actors in the field of experimentation.

Students and parents (or legal guardians) of each middle school in the 60 municipalities are informed by a letter at the end of 2020 by the referent and the principal/director of the component(s) (EMM or EMM + facilitation of access to the vaccination) deployed in their middle school between January and May 2021.

For the middle schools of the 30 municipalities randomized to groups 1 and 3 (benefiting from the 'Facilitation of access to vaccination' component), an information letter and collection of consent to vaccination are sent by the referent and/or each principal/school director, in January/February 2021, to the parental authority of each student enrolled in the school to propose the initiation of HPV vaccination at their child's school.

For each GP in the 45 municipalities randomized to groups 1, 2 and 5, an information letter on training (on motivational interviewing and the decision-aid tool) is sent by the PrevHPV SC. They are then contacted by telephone to check their eligibility and ask them to participate in the training.

The information intended for adolescents, their parents and GPs concerns the intervention carried out but also its evaluation methods (repeated cross-sectional survey KABP-6C).

7.3. Request for exemption from the collection of consent

The PrevHPV study is an interventional research in the field of public health, that is to say it is aimed at target populations (all adolescents attending middle school in the 90 participating municipalities and their parents) and not individuals specifically designated. The population-based nature of this research is difficult to reconcile with the collection of individual consent as requested by article L. 1122-1-1 of the Public Health Code. As a result, the CPP will be asked to be able not to collect individual consent from populations to participate in research, in accordance with article L. 1122-1-4 of the Public Health Code.

However, a letter (accompanied by an information note adapted according to the assigned group) will be sent to the families of all adolescents attending participating establishments via the schools in order to present the research to them.

7.4. Evaluation of the effect on knowledge, attitudes, beliefs and practices

The repeated cross-sectional survey (before – after) KABP-6C is carried out in the following populations:

- Adolescents (girls and boys), enrolled in Grades 8 & 9 classes in middle schools (public and private) located in the 90 participating municipalities.
- Parents of adolescents (girls and boys), enrolled in middle schools (public and private) located in the 90 participating municipalities.
- GPs practicing in municipalities in groups 1, 2, 5 and 6.

(i) KABP-6C survey among middle school students and their parents:

With authorization from the principals/directors of each middle school, the information and the invitation to the survey will be communicated to families through the middle school's usual means of communication. Parents may object to their child's participation. The T0 (before) and T1 (after intervention) questionnaires, self-administered and online, will be accessible via a specific research internet link.

The adolescent questionnaires will be administered in class under the supervision of a teacher or health professional. Each participant will be assigned an inclusion number, determined in advance by the middle school referent, to be entered in the T0 and T1 questionnaires which will make it possible to establish the link between the pre- and post-intervention questionnaires (T0 and T1). During the T0 survey, students will be asked to note this number in their correspondence notebook. A list of inclusion numbers and student names will be established for each class. All lists of the different classes will be kept in a closed envelope in the locked office of the research referent in the establishment, until they are transferred to the classes at T1. On this occasion, school staff present in class for the T1 questionnaire will be able to remind students of their inclusion number.

Parents will receive a survey invitation with the link to the research site. They will complete the T0 and T1 questionnaires at home, without the presence of children. Parents will be asked to enter their child's inclusion number (given in class and noted in the correspondence book), in order to be able to establish the family link while remaining anonymous. Parents will also be asked to note this inclusion number and keep it until the T1 survey.

The correspondence between inclusion number (which will make it possible to establish the link between T0 and T1, and between children and parents) and the identity of the participants will only be documented in the students' individual correspondence notebook and on the list kept for each class by the middle school's research referent. These lists will be destroyed after the questionnaires are administered to T1 in each class. Investigators will not have access to these lists.

(ii) KABP-6C survey among GPs:

For each of the municipalities in groups 1, 2, 5, and 6 the list of GPs practicing within each of them is established. These GPs are then contacted by telephone to ask them to participate in the KABP-6C survey. Each participant will be assigned an inclusion number which will make it possible to establish the link between the pre- and post-intervention questionnaires (T0 and T1).

7.5. Follow up

As part of this research, follow-up mostly concerns adolescents vaccinated in the middle schools. After the vaccine injection, each adolescent will stay with the doctor and nurse of the vaccination center for 15 – 20 minutes to check that they do not develop any adverse reactions. Beyond this duration, if an adverse effect occurs, the adolescent concerned notifies the school nurse (or the middle school research referent if it is not the school nurse) who is responsible for transmitting the information to the PrevHPV SC, which notifies the regional pharmacovigilance center of the region where the middle school is located.

The follow-up also covers participants in the KABP-6C survey, interviewed twice (T0 and T1). Students, parents of students and GPs agreeing to respond to the KABP-6C surveys will be asked to complete a standardized self-administered questionnaire twice, at T0 before the interventions and at T1 after the interventions.

There is no 'exit study visit' strictly speaking as part of this research.

7.6. Expected duration of participation

- Duration of participation of GPs: 10 months
- Duration of participation in middle schools (adolescents, parents, National Education professionals, SeSa Students): 10 months
- Total duration of the research: 10 months

(2020-2021 school year)

7.7. Stopping one's participation in the research

The cessation of participation of a person in the various components proposed as part of this research concerns:

- Adolescents whose holders of parental authority accept vaccination on the middle school site:
  - ✓ Holders of parental authority may withdraw their consent to vaccinate their child(ren), without justification, at any time between the transmission of the latter and the time of injection of the vaccine,
  - ✓ The doctor of the vaccination center may decide not to vaccinate the adolescent if he or she does not meet the inclusion/non-inclusion criteria which are re-checked at the time of injection of the vaccine, or if he or she considers that there is a particular risk for the adolescent.
- Parents of adolescents who agree to participate in the parent information meetings planned as part of the 'EMM' component may change their mind at any time between the date of acceptance and the time the dedicated meeting(s) is held.
- GPs who agree to participate in motivational interviewing training and the use of the decision-aid tool may change their mind at any time between the date of acceptance and the training.
- Participants in the KABP-6C longitudinal survey may stop and/or cancel their participation at any time.

The participation agreement of parents and GPs is an informal agreement, without signed consent as defined in article L. 1122-1-1 of the Public Health Code. This agreement may be withdrawn at any time before their participation (respectively in meetings and training). The only consent collected as part of this study is the consent of adolescents and their parents (holders of parental authority) to be vaccinated (see explanations in part 13.3).

7.8. End of research

7.8.1. Definition of end of research

The end of the research corresponds to the end of the completion of the KABP-6C questionnaires by adolescents, parents and GPs in the 90 participating municipalities (April-May 2021).

7.8.2. Description of the rules for permanently or temporarily stopping part or all of the research

In accordance with article R1123-26 of the Public Health Code, if the interventions have not started within two years following obtaining the favorable opinion of the CPP, the agreement of the committee will be considered lapsed and the research will have to be resubmitted to the authorities to be extended. This extension request must be accompanied by a letter justifying the delay compared to the initial forecast schedule.

During the research, if the defined research timetable is not respected, Inserm may decide to stop it if no other solution can be considered.

The sponsor and the competent authorities may interrupt the research for any other justified reason (major deviations from the protocol which do not guarantee the safety of participants, the quality of the data and the results of the research).

8. INTERVENTIONS EVALUATED IN THIS PROTOCOL

The three components of the intervention were developed: (i) based on a literature review carried out by the PrevHPV SC, (ii) as part of a co-construction process with the different stakeholders, and (iii) in light of the results of the diagnostic phase.

To date, the literature review has been carried out and the co-construction process is being finalized.

The three components are defined but may be refined/slightly modified depending on the results of the diagnostic phase. To date, they are as follows:

- **Education, Motivation, Mobilization (EMM)**

This component targets the parents of all adolescents enrolled in middle school and adolescents enrolled in Grades 8 & 9. It aims to provide scientifically reliable information on HPV infection and its vaccine. To do this, the promotion of existing websites on the theme (e-Bug, onsexpress.fr, stopHPV) will be carried out, and existing interactive tools and videos dedicated to adolescents will be considered. As tailored interventions are listed in the European Center for Disease Control's Catalog of Vaccine Hesitancy Interventions, adaptation of existing websites is encouraged.

This component firstly includes interventions with Grades 8 & 9 students. These interventions will be based on 3 main tools developed by the PrevHPV SC during the co-construction phase: a serious game, educational videos, and educational tools.

(i) The serious game: this is a game retracing the day of a teenager. During this day, the adolescent will meet individuals (teachers, friends, parents, school nurses) who will establish a dialogue around HPV-related infections and vaccination. This game will be produced as an application for smartphones and available on a website. It will be usable by adolescents, alone (inside or outside the middle school) or as part of class, but also by parents. This game will be partly co-created with adolescents, parents and teachers during the co-construction phase.

(ii) Educational videos of 10 to 12 minutes covering 4 themes: pathologies linked to HPV, risk of being infected by HPV, barriers to vaccination, advantages of being vaccinated. These videos will be constructed by health students during the co-construction phase, and will be the subject of a competition to select the best videos. They will be accompanied by explanatory sheets. The jury for this competition will be made up of the project steering committee: 3 videos will be selected. The call for competition will be distributed by the University College of Public Health Teachers, and Health student associations.

(iii) Educational tools and training intended for biology teachers and school nurses, including existing materials and materials created during the co-construction phase as part of the research (development of an interactive course that they can use with students).

This component also includes information meetings for parents who can come with their teenagers if they wish. These information meetings will be led by GPs, infectious disease specialists and/or gynecologists. Educational support will be co-created by the PrevHPV SC and can be used by speakers at these information meetings. In this context and when possible, we will call on health students integrated into the SeSa systems, particularly for interventions with students.

The implementation of the EMM component with adolescents and their parents will take place on the middle school site between 01/01 and 03/31/2021. The educational tools used during this implementation (serious games, videos and educational tools) will have been previously constructed by the PrevHPV SC in collaboration with the different target populations (adolescents, parents, school staff – co-construction phase).

- **Training of GPs**

The training of GPs covers the following three aspects: updated knowledge on HPV infection and its vaccination, short training in the use of motivational interviewing adapted to vaccination, training in the use of decision-aid tool developed by the PrevHPV SC as part of a co-construction process.

(i) The motivational interview style approach requires respectful and empathetic discussion about vaccination and helps build a strong relationship between parents/teens and the doctor. Parents and teens can freely discuss their concerns and ask questions about vaccination without feeling judged. From this type of doctor/patient relationship, the communication and information delivered by doctors are adapted to the needs of adolescents and parents since they are based on their own representations, concerns and questions. Doctors can identify and target the concerns or misconceptions of adolescents and parents about vaccination. They can thus provide adapted, personalized and therefore non-standardized information.

(ii) Decision aids are interventions that support patients by making their decisions explicit, providing information about options and associated advantages/disadvantages, and helping to clarify the congruence between decisions and personal values, in concordance with the motivational interview approach. This tool aims to provide in an impartial and objective manner the known and quantified scientific data on HPV (pathologies linked to HPV, risk of being infected, natural history, advantages of being vaccinated, adverse effects linked to vaccination). The tool personalizes the information according to its audience (a teenager will not necessarily have the same concerns as a teenager or even as parents). That is why, the decision aid tool (DA) is co-constructed with adolescents and parents (during the co-construction phase). This tool is also co-constructed with GPs in order to be able to integrate it more easily during the consultation. The International Patient Decision Aid Standards (IPDAS) will be used to design the decision aid (107).

The GP training being considered covers a total duration of four hours. It first includes up-to-date knowledge on HPV infection and vaccination, then an introduction to the use of the technique of motivational interviewing adapted to vaccination, ending with a presentation of the DA tool developed as part of the project to thus facilitate its appropriation. The training is constructed in several short modules and will be accessible online in the form of videos, slideshows, clinical vignettes and interactive modules to offer interview scenarios, follow-ups, questions and answers to patients' questions. To facilitate the adherence of GPs and the reproducibility of this training, we have chosen non-binding and non-face-to-face training, preferring the e-learning type (experience proves that doctors are increasingly resistant to face-to-face training, particularly in the event of geographical distance). To adapt to their different constraints, the training will be accessible at any time and GPs will be able to progress at their own pace. They will also have the opportunity to ask their questions on a discussion forum, moderated by team 2 (Pr. Serge Gilberg) of the PrevHPV SC. The training will be accessible online via the internet and there will be a version adapted for smartphones.

- **Facilitating access to vaccination**

For vaccination in middle schools in the 30 municipalities randomized to groups 1 and 3, the specialty chosen is Gardasil 9® from the MSD Vaccins laboratory which holds the monopoly to date and which is recommended by the HAS for first vaccinations. The choice was oriented towards this specialty because it is the one which offers the greatest protection against different oncogenic HPV serotypes (nonavalent vaccine protects against serotypes 6, 11, 16, 18, 31, 33, 45, 52 and 58), and it is therefore the one mentioned in the current vaccination recommendations (13).

In the 'facilitating access to vaccination' component, only the 1st injection is offered on the middle school premises. The reasons which motivated this choice are as follows:

(i) In 2018, the HPV VC in France was 29.4% for one dose in girls aged 15 and 23.7% for the complete regimen at 16 years (15), which shows that the main problem is the initiation of vaccination, since 80% of adolescents who initiate vaccination complete it (injection of a 2nd dose).

(ii) Planning two injections 6 months apart, preceded by the implementation of the EMM component, over a single school year was impossible since health students who participate in the EMM are only available beginning in January. In this context, carrying out the 2nd injection of the vaccine meant that some of the students (mainly those in Grade 9) had to be called back to the middle school even though they were no longer attending school there. This spread of the intervention over 2 school years raised numerous organizational difficulties.

However, measures will be implemented so that adolescents benefit from the complete vaccination schedule:

- mention of the 2nd injection to be carried out in both the written and oral information which will be provided by the doctor from the vaccination center (the date of the next injection will be mentioned orally and in post-vaccination letters) with provision of a prescription for the next injection,
- reminder letter as the 2nd injection approaches.

Once the list of the 30 municipalities benefiting from the 'Facilitation of access to vaccination' intervention has been identified (randomization in groups 1 and 3), contact is made with the DGESCO, the rectorate / diocesan management, the educational establishments, mayors, departmental councils and vaccination centers likely to intervene in educational establishments. A service contract is proposed between the University of Lorraine and the vaccination centers (with compensation for working time) so that they go to the middle schools of the different municipalities in question to vaccinate students whose parents consent to the vaccination.

The project managers of the PrevHPV team, in conjunction with each referent, are responsible for the practical organization of the “Facilitation of Access” intervention on site (date of the half-day(s) of vaccination, order and delivery of vaccines, etc.) and to create a link between middle schools and vaccination centers.

The PrevHPV project managers contact the middle school representatives designated during the “Education, Motivation, Mobilization” intervention and the vaccination center representatives. They agree on the date of vaccination according to the requests and availability of schools and vaccination centers (vaccination can take place over full days or several half-days). They define together the organization to be put in place and the equipment to be made available (various pre-vaccination/post-vaccination letters, familiarization with the equipment available on site: classroom, water point, bed, socket for the computer, table, chairs, emergency kit, catering for the workers if the intervention lasts one day, arrangements for handing over the students, etc.).

The steps of the process are as follows (see flowcharts below):

(i) In each middle school, the head of the school sends information to parents concerning the presentation of the PrevHPV project (accompanied by the information note specific to the interventions delivered). The middle school referent ensures that information on the vaccination day is disseminated within the school using the most appropriate channels.

(ii) PrevHPV project managers ensure the distribution of a pre-vaccination letter to schools. A letter containing the main information of the “Facilitation of access” component as well as consent to vaccination is sent to parents through the students, by email and/or made available on the ENT space (e- lyco or Ecole Directe). In this letter, mention is made of the characteristics allowing access to the vaccination offered, carried out by a vaccination center, within the school of the adolescent in question. These characteristics are those presented in the inclusion criteria (see inclusion criteria in the 'Facilitation of access' component, §5.2 Inclusion/non-inclusion criteria). If the adolescent is eligible for vaccination (compliance with all inclusion/non-inclusion criteria), then he or she can benefit from the first injection of the anti-HPV vaccine on the day scheduled at his or her middle school. If in any doubt, parents are encouraged to contact their child's GP or visit the website: http://vaccination-info-service.fr/.In the event that parents refuse to vaccinate their child, they can notify in writing directly on the consent document the reason for non-vaccination if they wish.

(iii) Parents' consent to their child's vaccination is sent to the school's PrevHPV referent. The PrevHPV referent of the middle school, with the possible help of the PrevHPV project manager of the geographical sector concerned, checks that all consents have been correctly completed (in particular the mandatory presence of the signatures of both parents (except in the event of the death of the parents or of child abandonment) and if necessary, they contact the parents to obtain the missing information. Once the consents for vaccination are complete, the middle school referent establishes the list of adolescents who can benefit from free vaccination within the middle school and keep consents to vaccination within the establishment in a lockable cabinet.

(iv) The PrevHPV project manager communicates the number of validated vaccination consents to the vaccination center in order to estimate the number of vaccines to order and bring for the vaccination day. Consents and the list of adolescents eligible for vaccination are kept at the middle school until the team from the vaccination center arrives.

(v) The logistical organization of vaccination on the site of each middle school is defined by the PrevHPV project managers in partnership with the vaccination centers and the schools. The PrevHPV project managers complete the poster informing of the date of the day or half-days of vaccination and send it to the schools so that they display it (This poster can be published in A5 format to be given to adolescents whose parents consent to their vaccination in order to remind them of the day of vaccination and the importance of bringing the health/vaccination record). Each school teaching staff is also informed of the day scheduled for vaccination.

(vi) On the day of vaccination, the vaccination center teams made up of at least: a doctor, a nurse (± the PrevHPV project manager) go to the middle schools with the vaccines previously received at the vaccination center and their emergency equipment in case of anaphylactic shock. The vaccination center team sets up in the provided premises. The students are sent to them according to a predefined order of passage in groups of two or three individuals in the room made available to the vaccination centers in accordance with the provisions previously established according to the organization of each establishment.

(vii) The doctor of the vaccination center verifies that the adolescent meets the inclusion criteria of the protocol, vaccinates and completes the health record.

(viii) On the nominative list of consents to vaccination, the vaccination center nurse or the PrevHPV project manager records whether the vaccination is carried out or not on a paper list.

(ix) All students who have received an injection remain under the supervision of the vaccination center doctor for 15 to 20 minutes before returning to class. During the post-vaccination surveillance period, a satisfaction questionnaire is given to adolescents who complete it on site. The nurse gives the student a post-vaccination letter in a sealed envelope intended for his or her parents in which it will be mentioned whether or not the vaccination has been carried out and the future injection(s) to be planned. (for parents who have consented but whose adolescent has not been vaccinated, a non-vaccination letter is sent to them). Subject to the acceptance of the parents of the vaccinated child (information formalized in the vaccination consents), a letter addressed to the adolescent's treating physician is sent directly either by the vaccination center or by the PrevHPV project manager of the geographical area concerned. In addition, parents can give a note to their community pharmacist if they wish to complete the pharmaceutical file.

In the post-vaccination information letters to parents and treating doctors, it will be specified that a second or even a third injection is necessary for the vaccination to be complete as well as the period at which it should be carried out. The prescription for the following injection will be made by the doctor at the vaccination center and provided with the other documents for parents.
At the end of the vaccination day, the satisfaction survey will be made available to the professionals of the school involved in the intervention in order to measure the possible incidence during the adolescents' school hours. The completed satisfaction questionnaires will be collected by the middle school referent.

Overall assessment of the vaccination day:

- Number of days organized
- Number of vaccinations requested
- Number of complete vaccination consents,
- Number of incomplete vaccination consents
- Number of vaccinations carried out
- Number and list of adverse effects
- List of technical or organizational problems
- Number of satisfaction surveys and results, collected from middle school students and professionals at the school.

(x) In the weeks following the vaccination day, at the initiative of the PrevHPV project manager, a meeting will be proposed with the referent and the director or principal of the school to summarize all of the EMM and Facilitation of access interventions. The summary will also be communicated to the vaccination center. The project manager will collect the satisfaction questionnaires from school teaching staff that same day from the middle school referent.

(xi) Depending on the age of the vaccinated adolescents, reminders are sent by email or postal mail to the adolescents' parents and the adolescents' treating physicians to inform of the need to complete the complete vaccination schedule:

- at 6 months for adolescents under 15 years old
- at 2 and 6 months for adolescents over 15 years old

- **Distribution of the implementation of interventions by The PrevHPV SC team**

The 'EMM' and 'Facilitation of access to vaccination' components will be implemented in the field by teams 1, 3, 4, and 5 who will distribute the 90 participating municipalities among themselves according to their geographical proximity.

The 'Training of GPs' component will be implemented in the field by team 2, with the help of team 3 (see part 15).

8.1. Vaccine logistics circuit

The purchase of vaccines administered as part of the PrevHPV - Facilitation of Access intervention (Gardasil9®, injectable suspension in pre-filled syringe Laboratoires MSD Vaccins) will be carried out in accordance with the procedures of the public procurement code, defined according to the overall cost of the vaccines. The purchase and supply of vaccines will be carried out and under the responsibility of one (or more) Pharmacy(ies) for In-house Use (PUI) of the participating regions, or failing that by a pharmaceutical service provider. For each middle school benefiting from the 'Facilitation of access' component, the middle school referent and the PrevHPV project manager of the region concerned will define the number of vaccines to be provided according to the feedback from vaccination consent forms. The vaccines will be sent to each vaccination center concerned a few days before the middle school vaccination day. On the day of the middle school vaccination, the mobile team from the vaccination center will transport the vaccines previously received. All vaccine deliveries (between the supplier and the PUI, between the PUI and the vaccination centers, between the vaccination centers and the middle schools) will respect the cold chain.

There will be no specific packaging and/or labeling of the vaccines as the vaccine itself is not the subject of the research. The present research aims to evaluate the effect on vaccination coverage of a change in access to vaccination, the vaccine in question having a marketing authorization for the population targeted by the study.

The entire drug circuit (purchase, supply to PUIs and vaccination centers, storage, transport to middle schools) will comply with current pharmaceutical regulations.

The preparation and organization of vaccination days is the subject of a procedure. The vaccine circuit is currently being developed.

8.2. Allocation modalities

8.2.1. Randomization

Two successive randomizations are implemented:

(i) A first randomization aimed at selecting 409 municipalities from the list of 2049 eligible municipalities, identified from the data.education.gouv.fr site. For reasons of feasibility, different sampling fractions were applied for the municipalities of the departments in which the SC teams are located (1/2), the municipalities of other departments but belonging to the regions in which the SC teams are located (1/5), regional municipalities in which SC teams are not located (1/8).

(ii) A second randomization aimed at assigning the assignment group (among the 6 in the research) to each of the 90 municipalities participating in the research (participation agreement for all middle schools). The 90 municipalities will be randomized concomitantly ('all at once').

These 2 randomizations are stratified on the department and the ecological indicator of deprivation (FDep) dichotomized into 2 classes (< or > median of eligible municipalities in the department). They are carried out by the EA APEMAC (University of Lorraine), using the SAS software version 9.4 (Proc RandomSampling).

8.2.2. Authorized and prohibited treatments during research including rescue treatments

No treatment is prohibited during research.

During the half-days of vaccination on the middle school sites, the teams (doctor and nurse) from the vaccination center will travel with a first aid kit, containing medications and medical devices allowing them to deal with a possible anaphylactic or other reaction. adverse event considered 'serious'. The severity and appropriate management of the event will be left to the discretion of the vaccination center doctor.

9. SAFETY

9.1. Definitions

An adverse effect is defined as any harmful and unwanted manifestation likely to be linked to the handling or consumption of a product, a substance or occurring during an act of care.

An adverse reaction meets the definition of “serious” if it:

- causes death;
- endangers the life of the participant (effect during which the subject risked death; it does not designate an event/effect which hypothetically could have caused death if it had been more serious);
- requires hospitalization or extension of hospitalization;
- causes significant or lasting disability or incapacity;
- results in a congenital anomaly or malformation;
- is a “significant medical event” (event considered by the investigator to be medically important and which may endanger the subject or require intervention, medical or surgical, to prevent one of the characteristics/consequences mentioned above. Examples: treatment intensive care in hospital emergency rooms or at the research participant's home for allergic bronchospasm, seizure or coagulation disorders).

9.2. Responsibilities of the investigator

The investigator immediately declares to the sponsor any serious adverse effect likely to be linked to the research procedures and any new fact, that is to say any new safety data which may lead to a reassessment of the benefit/risk ratio of research, or which might be sufficient to consider changes to the research documents.
To do this, he must immediately send a copy of the document detailing the circumstances of the serious adverse effect or the new fact to the addresspharmacovigilance.prc@inserm.fr

Any other adverse effect likely to be linked to a product taken by the participant must be declared by the investigator to the competent structures in accordance with the applicable regulations (http://social-sante.gouv.fr/grands-dossiers/signalement-sante-gouv-fr in France).

9.3. Potential risks of research and what to do in the event of an adverse effect

Given the category of research, RIPH2 Research with Minimal Risks and Constraints, the foreseeable risk added by the research is negligible (self-administered questionnaires), no serious adverse event linked to the acts and procedures of this research is predictable.

10. DATA COLLECTION AND PROCESSING

The processing of the personal data of persons participating in this research has the sole purpose of carrying out the research. This processing includes the management of data relating to people taking part in research, with a view to enabling the collection, entry, checking of validity and consistency and statistical analysis of the data collected during the research.

As part of this research, two types of databases must be considered:

(i) A research management base which will allow the practical carrying out of research in the field and which includes a certain number of nominative data. This base should mainly include the following variables: name of participating municipalities and randomization group; name of the middle schools, the principal/director, the nurse/doctor, the PrevHPV referent within each middle school; name of GPs and GPs participating in the training of each municipality; name and address of middle school students who have accepted vaccination on the middle school website (for sending the 6-month reminder, etc.). For monitoring within the framework of the KABP-6C survey more particularly,

    The research management base will be accessible to the teams responsible for implementing the intervention in the field, that is to say teams 1, 2, 3, 5 and 7. It will be shared between these teams (computer network) and within each of the 5 teams, only authorized people have the right of access with identification of authorized users.

(ii) A research database which contains all the data necessary to exploit the results corresponding to the research objectives, but no nominative data. The data collected will not identify the person who participated. Furthermore, there is no common variable allowing the link between the

research management base and the database. The accesses and the circuit of this data of this last base are described below. In the same way as for the management database, only authorized people have the right of access with identification of authorized users, in accordance with the operating procedures defined within the framework of the research.

The paragraphs below regarding the research database.

10.1. Description of collected data

The data collected to meet the objectives of this research are as follows. They were defined with a care for minimization:

a) The main characteristics (number of middle schools, department, FDep, medical density) of the participating municipalities, as well as the participating middle schools (size of the middle school, REP(+) middle school or not, number of FTE school nurses/doctor, urban sector or rural).

b) The main characteristics of GPs (age group, gender, seniority and area of practice, mode of practice, sector of practice).

c) The data completing the SNDS data for the calculation of the vaccination coverage rate (1 dose, 2 doses) at the different times (before intervention, 2, 6 and 12 months after the end of the intervention) i.e., for each participating municipality, injection statistics in family planning and education centers and vaccination centers, as well as the number of vaccines administered in each middle school in groups 1 and 3 during the intervention.

d) Data on individual characteristics, knowledge, attitudes, beliefs (opinions on the effectiveness, safety or usefulness of the vaccine), practices related to vaccination in general and HPV vaccination, antecedents of hesitancy vaccination, intention to vaccinate among adolescents, their parents, and GPs, and declared vaccination status.

e) Data on health students' knowledge and vaccine hesitancy.

f) Data on the satisfaction of the different target populations in relation to the actions implemented (SeSa students, middle school professionals, GPs/training, adolescents and parents/e-health tools made available).

g) Data on the number of middle schools/municipalities asked to participate in the research and the number who accepted, the number and nature of the actions carried out for each of the 3 components, the participation of the target populations in the various actions carried out, possible adaptation of actions (compared to what was planned) according to the context and/or local constraints.

h) Cost data: resources associated with each of the 3 components (design + implementation), collected from the project teams in charge of their development (for the tools), middle school referents, project managers and training centers. vaccination (for their implementation).

The data collected in b, d, e, and f are collected individually but anonymously, that is to say without collection of indirectly identifying variables.

Justification of the data collected:

Data	Rationale
Characteristics of municipalities and middle schools	Description of the municipalities / middle schools concerned by the 'EMM' and 'Facilitation of access' components of the intervention<br>Response to secondary objective 2
Characteristics of GPs	Description of the population concerned by the 'Training of GPs' component
VC calculation data (in addition to SNDS data)	Estimation of the overall VC rate (Response to the primary objective)
Data on individual characteristics and knowledge, attitudes, beliefs, practices, history of vaccine hesitancy, intention to vaccinate and reported vaccination status	Evaluation of the impact of the different components in the different clusters using pre- and post-intervention questionnaires (Response to secondary objective 1)
Satisfaction data from different populations	Assessment of the satisfaction of different populations with the different components of the intervention and identification of levers and barriers (Response to secondary objective 5)
Implementation data	Evaluation at the individual and collective level of the implementation of the intervention and identification of levers and barriers (Response to secondary objective 5)
Cost data	Evaluate the efficiency of the different components of the intervention and the cost of these different components alone and combined (Response to secondary objectives 3 and 4)

No individual health data, other than whether or not someone is vaccinated, is collected as part of this research.

Personal data (eligibility criteria and contact details) concern GPs in the 90 municipalities and participants in the KABP-6C survey.

Each participating municipality and middle school will be assigned a 'municipality code' and a 'school code' respectively. These codes will only be known by the PrevHPV SC project teams and will be linked in a file dedicated to a URL address allowing access to the questionnaires (KABP-6C questionnaire). At no time will the names and contact details of middle schools be recorded in a file containing the data collected from the populations studied. At no time will the names and contact details of participants in the KABP-6C survey be recorded in a file containing the data collected from participants.

10.2. Source data and documents

Source data is all the information contained in original documents, or in authenticated copies of these documents, which relate to clinical examinations, observations or other activities carried out as part of research involving the person human and which are necessary for the reconstruction and evaluation of research. The documents in which the source data are recorded are called source documents, regardless of the medium used (paper, electronic, etc.).

The data a) are collected on a specially dedicated computer database and are shared by all the PrevHPV

SC teams. These data are public data, accessible via online statistics. They will be verified with the principals/directors of the middle schools.

Data b) are collected on a specially dedicated computer database and are shared between team 2 and team 3. They will be collected by teams 2 and 3 by interviewing the GPs participating in the training.

Data c): (i) SNDS data (for participating municipalities: reimbursement of a HPV vaccine for a young person aged 11 to 14, by sex, and prescribing health professional) will be made available to team 8 (dedicated data access portal) after contracting with the CNAM and obtaining authorizations. (ii) data from family planning and education centers, vaccination centers and number of vaccines used for research will be collected by teams 1, 3, 4, and 5 for each geographical area that each team is in charge of and will be transmitted to team 8 for the analysis of the main objective and to team 6 for the evaluation of efficiency.

Data d) are collected via the KAPB-6C self-questionnaires administered online to the different target populations on a database generated by team 7.

Data e) are collected via self-questionnaires administered online to SeSa students on a database generated by team 5.

Data f) on satisfaction with the interventions are collected via online or paper self-questionnaires. (SeSa students, middle school professionals, adolescents and parents will be managed by teams 4 and 5; GPs by team 2).

Data g) and h) are collected as the intervention components are designed and implemented by the SC project teams on an electronic CRF and will be transmitted to team 1 for data g) and to team 6 for data h).

The online self-administered questionnaires (KABP-6C) will be developed using RedCap® software. The data will be stored with the inclusion number but without other identifying information in the RedCap® collection databases, which will allow an anonymous survey to be carried out. After closure, the data from each survey will be extracted into a database and deleted from the RedCap® software.

10.3. Data circuit

Data	Source	Team(s) in charge of recovering data	Team(s) to whom the data is transmitted (or accessible) for analysis
a)	Public data	1	All SC teams
b)	Interviewing GPs	2 and 3	2
c)	SNDS<br>Other than SNDS	8<br>1, 2, 3, 4, 5	8 and 6 (efficiency)
d)	Online self-questionnaires	7	7
e)	Online self-questionnaires	5	5
f)	Online self-questionnaires	2, 4, 5	2, 4, 5
g)	Interviewing project teams on site	1, 2, 3, 4, 5	1
h)	Interviewing project teams	1, 2, 3, 4, 5	6

Each type of data, a) to h) above, will be hosted on computers of the teams to whom the data is transmitted for analysis. They will be accessible to members of the the PrevHPV SC according to the rules of the consortium.

The data collected cannot be transmitted to third parties outside the 8 PrevHPV SC teams.

The data management, the statistical analysis plan, the database freezing and the regular backup of the data will be carried out according to the procedures in force within each team which is in charge of the statistical analysis for the part which concerns it.

10.4. Conservation of research documents

Documents relating to research are archived in accordance with current regulations.

The sponsor and the PrevHPV SC keep the documents relating to the research, which are specific to them until the end of the statistical analyzes (expected duration: 5 years after the freezing of the database) making it possible to meet the objectives of the research (conservation of the active base), then beyond for a total period of 15 years (conservation in archiving).

No movement or destruction can be done without the agreement of the sponsor. At the end of the regulatory archiving period, the sponsor will be consulted for destruction.

All data, documents and reports are subject to audit or inspection.

11. STATISTICAL ANALYSIS

11.1. Responsible for statistical analysis

Responsible for the analysis relating to the primary objective and secondary objective 2 (evaluation of effectiveness): Professor Bruno Giraudeau (Team 8)

Responsible for the analysis relating to secondary objective 1 (assessment of knowledge, attitudes and practices, antecedents of vaccine hesitancy, KABP-6C): Dr Judith Mueller (Team 7)

Responsible for the analysis relating to secondary objectives 3 and 4 (evaluation of efficiency and budgetary impact): Professor Karine Chevreul (Team 6)

Responsible for the analysis relating to secondary objective 5 (evaluation of the implementation): Professor Nathalie Thilly (Team 1)

11.2. Sample size calculation

The sample size will depend on the number of participating middle schools, which is not known at this stage. This is why at this stage, we will consider the situation of a single middle school per municipality. The calculation below is therefore conservative.

According to government statistics, the number of students per middle school is on average 466. Assuming one middle school per cluster, the average size of the cluster would therefore be 466 adolescents aged 11 to 14. Twenty percent of middle schools having fewer than 300 students and 13% having more than 700, we retain a coefficient of variation of 0.5 in the size of the clusters.

The intra-class correlation coefficient is set at 0.05.

The current prevalence of initiation of vaccination from 11 to 14 years old is 16.1% among girls and 0% among boys due to the date of application of the HAS recommendation on the vaccination of boys. Girls representing 48.85% of the population in this age group, the prevalence among 11-14 year olds is 7.86%. The objective of the intervention is to increase this prevalence by at least 10% (based on data published in the literature). Considering two groups, we would need 15 clusters per group to reach a power of 90% and considering an alpha risk of 5%.

This research will include 6 groups, and the analysis will be carried out using a modeling approach in which several parameters will be tested: effects of interventions, and potential interactions between interventions. It is not planned to make multiple 2 to 2 comparisons. Therefore the alpha risk has not been corrected.

In the end, knowing that 6 groups will be formed, we plan to include a total of 90 municipalities (15 per group). This corresponds to an expected sample of 41,940 adolescents aged 11 to 14, under the assumption of a single middle school per municipality.

11.3. Description of the statistical analysis plan

A data management and statistical analysis plan will be drawn up by the PrevHPV SC before the start of the research.

Qualitative variables are expressed as counts and percentages, and quantitative variables as means, standard deviations, medians and interquartile ranges.

All analyzes are carried out on an intention-to-treat basis. No interim analysis (effectiveness or futility) is planned before the end of the intervention.

11.3.1. Analysis of the primary outcome

The prevalence of vaccination (at least 1 dose received and complete vaccination) among adolescents aged 11-14 is calculated (from SNDS data, supplemented where appropriate with data from vaccination centers, family planning centers and vaccines delivered in middle school) at the different measurement times (before intervention, 2 months, 6 months and 12 months after intervention) and in each of the six study groups. A calculation of this prevalence according to the sex and deprivation level of the municipality of residence of the adolescents is also planned.

Concerning the analysis of the primary outcome (vaccination coverage rate), an analysis at the cluster level will be carried out, that is to say that the unit of analysis will be the municipality and not the adolescent who is nested in his or her municipality. The data will be collected at the municipality level. Each municipality (= cluster) will be weighted by its size. A modeling approach will be used, carrying out a global analysis of the 90 randomization units. The model will be a linear model in which we will consider 3 fixed effects (one per component) and potential interactions between component. The baseline VC will be considered as an adjustment variable.

The evolution of the VC over time (M2, M6, M12) will also be modeled by considering a longitudinal model, and adjusting for the baseline VC rate.

Subgroup analyzes will be carried out according to sex, deprivation level (FDep) and considering a modeling approach with interaction terms.

Depending on the quality of the data reported by vaccination centers and family planning centers, sensitivity analyzes will be carried out to take into account only SNDS data and the number of vaccines delivered in middle school.

The analyzes are carried out using SAS software version 9.4 or later (SAS Institute Inc.) or R, and considering two-sided statistical tests and a level of significance of 5%.

11.3.2. Analysis of secondary outcomes

(i) Analysis of secondary objective 1

For the analysis of this objective, people with a positive change in intention/vaccination will be identified (change from “no” for both at T0 to “yes” for one of the two at T1).

The variables corresponding to the KABP-6C items will be created in particular by calculating knowledge scores and Likert scale medians.

For an analysis of T0, the percentages and medians of the items will be presented by intervention group, namely:

a. By EMM groups 0 and 1, among participants without vaccination campaign, without taking into account the GP intervention
b. By school interventions (campaign + EMM vs. without campaign or EMM), without taking into account the GP intervention
c. By GP groups 0 and 1, among participants with EMM, without taking into account the vaccination campaign intervention

For an analysis of the main effects, the percentages of people with change and the medians of the differences will be presented by group (see a., b., and c.).

The differences will be tested first by suitable non-parametric tests (Fisher's and Wilcoxon, ranksum).

Multilevel models with a binary or continuous (linear) dependent variable will then be constructed in order to estimate the effect of interventions alone or in interaction, while taking into account intraclass correlation effects (cluster randomization). The interactions to be studied as a priority are:

- between the GP intervention and the school intervention (EMM+campaign),

- between the vaccination campaign intervention and the GP intervention (only possible in combination with EMM)
- between the EMM intervention and the GP intervention (only possible in the absence of a vaccination campaign)

For an analysis not taking into account individual evolution between T0 and T1, the effect of each intervention (alone or in interaction) will be estimated from a difference-in-difference model, i.e. including an interaction between the intervention group (among the 6 defined, with the control group as reference) and the temporal indicator "after" (vs. before) intervention.

Variations in the impact of intervention components will be explored by analyzes stratified by individual characteristics and KAP-6C (at T0). In order to test for significant differences between subgroups, we will examine 95% confidence intervals. In general, we will neither apply predefined statistical significance levels nor correct for multiple testing, but report exact P values. Interaction terms may be included in the models, if indicated.

Models including individual characteristics will be built for an analysis of the determinants of vaccination intentions.

Other analyzes will focus on the change from "intention without vaccination" to "vaccination declaration"; and on the association between individual characteristics, KAP-6C items at T0 or T0-T1 change.

Statistical analyzes will be carried out using STATA software.

(ii) Analysis of secondary objective 2

A descriptive analysis of the outcomes at their different measurement times is carried out by quantile of social deprivation (FDep) in order to study the existence of a social gradient, which could be the sign of a worsening or of an improvement in social health inequalities with regard to HPV vaccination. The FDep is also introduced as an adjustment variable in the multivariate regression models (see 10.3.1) in order to highlight an association of the level of deprivation with the effectiveness of the intervention, all things being equal. Interactions of the variables of interest with deprivation will also be tested in the models.

(iii) Analysis of secondary objective 3: Evaluation of efficiency

A cost-effectiveness analysis is carried out in accordance with the recommendations of the HAS (104). The perspective retained is that of all payers, the HPV vaccine having a significant out-of-pocket cost. The time horizon is set at 2 months after the end of the intervention in the main analysis. Secondary analyzes will also be carried out at longer time horizons (6 and 12 months after the end of the intervention). In agreement with the HAS, no discount rate will be applied. All adolescents living in municipalities will be included in the economic evaluation. The effectiveness criterion will be the increase in the prevalence of HPV vaccination (1 dose), and the costs will include the costs of the vaccine, consultations, and interventions implemented. An incremental cost-effectiveness ratio will be calculated to evaluate the incremental costs needed to gain 10% additional vaccination coverage. Deterministic and probabilistic sensitivity analyzes will be performed to assess the uncertainty surrounding the results.

The economic evaluation will focus on the intervention as a whole and on each of its components.

(iv) Analysis of secondary objective 4: budgetary impact analysis

If the intervention (or one of its components) is cost-effective (objective 3), a budgetary impact analysis will be carried out to assess the financial consequences and health gains associated with its generalization to the entire target population. This analysis will be carried out from the perspective of the statutory health insurance and considering a 5-year time horizon, in accordance with French recommendations (105).

The target population is made up of all adolescents aged 11 to 14 and two scenarios will be compared in this population:

- A first scenario in which no component is implemented to improve the vaccination coverage rate (current situation),
- A 2nd scenario in which 1, 2 or all 3 components (depending on the results of the cost-effectiveness study) of the intervention evaluated are implemented at the national level.

The budgetary impact analysis will compare the annual costs and health benefits of each of the two scenarios. Deterministic sensitivity analyzes will also be carried out to assess the uncertainty surrounding the results.

(v) Analysis of secondary objective 5

Descriptive analyzes will be carried out to present the participation rate of municipalities, the number and nature of actions carried out for each of the 3 components, the participation rate of target populations in the different actions carried out, the possible adaptation of actions (compared to what was planned) depending on the context and/or local constraints.

12. CONFIDENTIALITY

12.1. Terms of confidentiality with respect to individuals

Self-questionnaires are completed online (KABP-6C questionnaires and general practitioner satisfaction questionnaires) and on paper (other satisfaction questionnaire). It will be done once, at each measurement time.

A URL link will be created for each participating middle school (adolescent questionnaires, parents, middle school professionals, SeSa students) and each municipality (general practitioner questionnaires) and will allow teams 5 and 7 to follow the evolution of the completion rate and to send reminders the middle schools/municipalities for which low participation is observed.

All research databases do not contain information allowing the respondents/participants to be directly or indirectly identified.

12.2. Terms of confidentiality with respect to the research

Access to the data will be direct in the event of monitoring, audits ordered by Inserm, or inspections possibly carried out by the CNIL or any other competent authority when applicable.

13. COMMUNICATION

The PrevHPV research (experimental phase) will be recorded on the site www.clinicaltrials.gov after obtaining the agreement of the CPP.

13.1. Conditions relating to the publication of results

All data collected during this research are the property of the sponsor and cannot be communicated under any circumstances to a third person without the written consent of the sponsor.

The results are published after final analysis in the form of scientific articles in peer-reviewed journals, presented at national and international conferences. The rules for publication or communication (oral or written) based on PrevHPV research data and the intellectual property rules are defined in the consortium agreement which brings together the different SC teams. Publications will comply with international recommendations: “UniformsRequirements for ManuscriptsSubmitted to BiomedicalJournals” (http://www.cma.ca/publications/mwc/uniform.htm).

Any publication will also follow the rules present in the publications charter defined by AVIESAN. The mention of the origin of the financing, the authorizations of the competent authorities, the consent of the participants will appear in the acknowledgments according to the following model:

/Ethics statement //This study is part of the randomized controlled trial PrevHPV sponsored by Inserm. It was granted approval by local Ethics Committee or “Comité de Protection des Personnes” on ---****DATE**---, and registered in a public trials registry (****CT XXXX**).

In compliance with the commitments made by Inserm and regulatory obligations, the results will be published on the public site on which the research was recorded.

13.2. Conditions relating to the summary of the final report and the final report

Once the research is completed, a final report of the results is produced within one year of the end of the research in all countries where it was conducted.

The final research report is a written document, sufficiently detailed to allow you to understand the progress of the research and to make an objective judgment on the quality of the data from this research. It is written, in collaboration, by the coordinating investigator and the biostatistician of this research and submitted to all investigators for their opinion. Once a consensus is obtained, the final version is endorsed by the signature of each of the investigators and made available to Inserm and the CPP. It will only be sent to the ANSM at its request.

This report includes a summary of the results written according to the reference plan of the competent authority. The summary is validated and transmitted by Inserm to the CPP and the ANSM according to the conditions

established for interventional research on category 2 humans. This transmission will be carried out within one year following the end of the research.

The final report of the research, supplemented by a discussion on the recommendations of the PrevHPV SC in terms of organizational development in order to improve the acceptability of anti-HPV vaccination in France, will be sent to the health authorities involved in the development / evaluation or financing of the project.

13.3. Methods for informing people participating in research about its overall results

At the end of the research, the overall results of the research will be sent: (i) to the GPs who participated in the training planned as part of the intervention evaluated, (ii) to the principals/directors of the middle schools established in the 90 participating municipalities, (iii) to the mayors of 90 participating municipalities. The representative of each participating middle school will then be able to disseminate the results to their staff, to the students enrolled during the intervention and to their parents (the contact details of the students are always available from one year to the next).

13.4. Conditions relating to press communication

The articles and abstracts as well as oral communications resulting from this research will be sent before publication to the Clinical Research Center and the Department of Scientific Information and Communication (DISC).

14. PROTECTION OF INDIVIDUALS

14.1. Ethical justification of the protocol

The research is carried out in compliance with the provisions set out in article L.1121-2 of the Public Health Code.

14.2. Suitability of location for research

In accordance with article L1121-13 of the Public Health Code, the research will be carried out in a location with human, material and technical resources adapted to the research and compatible with the safety requirements of the people involved.

The only research-related risk is related to the injection of the HPV vaccine. As indicated in the 'Research rationale' section, the current data on the safety of this vaccine are very reassuring, after 10 years of pre-MA studies, 15 of real-life surveillance and 270 million doses injected in 2017. Risk of anaphylactic reaction is estimated at 1.7 cases per million doses. Discomfort is also possible, but the consequence of anxiety/stress due to the injection of a product (23). During the half-days of vaccination on the middle school sites, the teams (doctor and nurse) from the vaccination center will travel with a first aid kit, containing medicines and medical devices allowing them to manage a possible anaphylactic reaction or other adverse event.

14.3. Ethical and regulatory provisions

The research will be carried out in compliance with current French regulations, in particular the provisions relating to research involving humans provided for in articles L 1121-1 et seq. of the Public Health Code, the Bioethics laws, the Informatics and Freedom law, the General Data Protection Regulation, the Declaration of Helsinki, and this protocol.

The PrevHPV SC and the coordinating investigator undertake to conduct the research in accordance with these ethical and regulatory provisions. They are aware that all documents as well as all data relating to research may be subject to audits and inspections carried out in compliance with professional secrecy and without medical confidentiality being able to be opposed. The PrevHPV SC acknowledges that the research results are the property of Inserm, the research sponsor.

According to article 5 of the decree of April 12, 2018 establishing the list of research mentioned in 2° of article L.1121-1 of the Public Health Code, are deemed to be interventional research on humans (RIPH) category 2 research relating to a program, an action or a public policy aimed at modifying the practices or behavior of healthy or sick people and likely to have an influence on their health. The PrevHPV research corresponds to this definition and therefore falls within the framework of an RIPH2.

14.4. Committee for the Protection of Persons (CPP)

Before carrying out the research, the sponsor submits the PrevHPV project to the opinion of a committee for the protection of persons designated at random under the conditions provided for in article L. 1123-14 of the Public Health Code and will provide it with for this all the necessary information.

The research can only begin when Inserm has been informed of the unreserved favorable opinion delivered by the CPP regarding the protocol submitted subject to obtaining any other authorization necessary for the implementation of the research. This notice will include the title and number of the protocol assigned by the sponsor, the documents examined, as well as the date of its examination and the list of members of the CPP who participated.

The sponsor will inform the CPP of all subsequent amendments.

14.5. ANSM

Authorization from the ANSM is not required for this study. The ANSM will be informed by the sponsor of the start of this study.

14.6. CNIL

This research cannot begin until authorization has been obtained from the National Data Protection Commission (Cnil).

14.7. Insurance and financing

Inserm, as sponsor, has taken out a civil liability insurance contract for the entire duration of the research under the number XXX, in accordance with French legal and regulatory provisions on category 2 research.

15. GOVERNANCE AND COMMITTEE

There will be no independent oversight committee due to the negligible risks expected from this research.

A consortium made up of the eight research teams working on the PrevHPV project was formed (see table below).

**1**		EA 4360 APEMAC - University of Lorraine<br>9 BC de la Forêt de Haye - BP 20199 - 54505 VANDOEUVRE LES NANCY Cedex<br>Scientific manager and principal investigator: Pr THILLY Nathalie Tel: 03.83.15.72.76 - Email:n.thilly@chru-nancy.fr
**2**		Department of General Medicine - University of Paris - 24 rue du Faubourg Saint-Jacques - 75679 PARIS Cedex 14<br>Scientific referent: Professor GILBERG Serge<br>Tel: 01.44.41.23.63 - Email:sergegilberg@gmail.com
**3**		Interuniversity Psychology Laboratory - UFR Human and Social Sciences - Université Grenoble Alpes<br>BP 47 - 38040 GRENOBLE Cedex 9<br>Scientific manager: Dr GAUCHET Aurélie<br>Tel: 04.76.82.58.67 - Email:aurelie.gauchet@univ-grenoble-alpes.fr
**4**		CRCDC Pays de la Loire<br>5 rue des Basses Fouassières - 49000 ANGERS<br>Scientific manager: Dr LE DUC-BANASZUK Anne-Sophie Tel: 02.41.05.06.67 - Email:as.banaszuk@depistagecancers.fr
**5**		Campus Santé Innovations - Faculty of Medicine Jacques Lisfranc<br>10 rue de la Marandière - 42270 SAINT-PRIEST-EN-JAREZ<br>Scientific manager: Dr GAGNEUX-BRUNON Amandine<br>Tel: 04.77.12.03.56 - Email:amandine.gagneux-brunon@chu-st-etienne.fr
**6**		INSERM UMR 1123 ECEVE, UFR Paris Diderot, 75010 PARIS<br>Scientific manager: Pr CHEVREUL Karine<br>Tel: 01.40.27.41.48 - Email:karine.chevreul@urc-eco.fr
**7**		Institut Pasteur - 25 rue du Dr Roux - 75724 Paris cedex 15 Scientific manager: Dr MUELLER Judith<br>Tel: 01.45.68.82.17 - Email:Judith.MUELLER@ehesp.fr
**8**		University Hospital of Tours - Bretonneau Clinical Investigation Center - 37044 Tours cedex 9<br>Scientific manager: Professor GIRAUDEAU Bruno<br>Tel: 02.34.37.96.57 - Email:bruno.giraudeau@univ-tours.fr

The governance of the PrevHPV project is generally structured around 2 committees:

- Steering committee called SC
- Monitoring committee.

15.1. Steering Committee (SC)

This operational committee is made up of the Scientific Managers of each party and a representative of the sponsor. It meets monthly to develop, implement and monitor the progress of the research.

15.2. Monitoring committee

The Monitoring Committee's mission is to monitor the execution of the Agreement, and in particular the progress of the project's work; to propose solutions in the event of an execution problem.

The Monitoring Committee constitutes a privileged body for communication between the Parties of all information, whether of a technical, industrial, commercial or other nature.

16. QUALITY ASSURANCE

16.1. Description

The research is supervised according to standard Inserm procedures. All specific research procedures must be validated by the sponsor.

The role of quality assurance is to guarantee the safety of people who take part in research involving humans and to ensure the credibility of the data resulting from this research and their recognition by the medical and scientific community.
The investigator is the guarantor of the quality of the research.

16.2. Monitoring (research quality control)

Given the low risk of this research, there are no plans to establish a monitoring plan. However, regular reviews will be carried out (at a pace to be defined) between the PrevHPV project managers (in conjunction with the referents in the middle schools) and a representative of the sponsor in order to ensure monitoring of the research and to study any discrepancies. by comparing achievements and forecasts of actions.

17. SUBSTANTIAL CHANGES TO THE PROTOCOL

Any request for modification of the research in relation to the initially authorized research project must be submitted, by the coordinating investigator (Pr. Nathalie THILLY), for opinion, to the sponsor.
Submission procedures are available on the Inserm intranet.
After a favorable opinion, Inserm will implement the regulatory administrative procedures necessary to obtain approval of these substantial modifications by the CPP and/or the competent administrative authority.

18. REFERENCES

1. Bansal A, Singh MP, Rai B. Human papillomavirus-associated cancers: A growing global problem. Int J Appl Basic Med Res 2016; 6(2):84-9.

2. GLOBOCAN Cancer Fact Sheets: Cervical cancer [Internet] [cited 2018 Jan 28]. Available on :http://globocan.iarc.fr/old/factsheets/cancers/cervix-new.asp

3. Hamers FF, Woronoff AS, French network of cancer registries Francim. Cervical cancer in France: trends in incidence and mortality until 2018. Bull EpidémiolHebd 2019;(22-23):410-6.

4. Garland SM, Kjaer SK, Muñoz N, Block SL, Brown DR, DiNubile MJ, et al. Impact and Effectiveness of the Quadrivalent Human Papillomavirus Vaccine: A Systematic Review of 10 Years of Real-world Experience. Clin Infect Dis Off Publ Infect Dis Soc Am 2016;63(4):519-27.

5. Luostarinen T et al. Vaccination protects against invasive HPV-associated cancers. Int J Cancer. 26 Dec 2017.

6. Joura EA, Giuliano AR, Iversen OE, Bouchard C, Mao C, Mehlsen J, et al. A 9-valent HPV vaccine against infection and intraepithelial neoplasia in women.N Engl J Med. 2015 ; 372(8):711-23.

7. HCSP. Prevention of HPV infections: role of the Gardasil 9® vaccine [Internet]. Paris: High Council of Public Health; Feb 2017 [quoted Feb. 13 2019]. Available at: https://www.hcsp.fr/explore.cgi/avisrapportsdomaine?clefr=603.

8. WHO | WHO Director-General calls for all countries to take action to help end the suffering caused by cervical cancer [Internet]. WHO. [cited Dec 20 2018]. Available on :http://www.who.int/reproductivehealth/call-to-action-elimination-cervical-cancer/en/.

9. HCSP. Vaccination against human papillomaviruses 16 and 18 with a bivalent vaccine [Internet]. Paris: High Council of Public Health; 2007 Dec. [quoted Feb. 21 2018]. Available at: https://www.hcsp.fr/explore.cgi/avisrapportsdomaine?clefr=19.

10. HCSP. HPV infections in young girls: review of the vaccination age [Internet]. Paris: High Council of Public Health; 2012 Sep [cited Feb 21 2018]. Available at: https://www.hcsp.fr/explore.cgi/avisrapportsdomaine?clefr=302.

11. HCSP. HPV infections: new vaccination schedule for the Gardasil® vaccine [Internet]. Paris: High Council of Public Health; 2014 March [cited Dec 19 2018]. Available at: https://www.hcsp.fr/explore.cgi/avisrapportsdomaine?clefr=416.

12. Sonawane K et al. Prevalence of human papillomavirus infection by number of vaccine doses among US women. JAMA Netw Open 2019;2(12):e1918571.

13. Vaccination recommendations against human papillomavirus infections in men[Internet].[CitedDec.19, 2018].Availableon: https://www.hcsp.fr/explore.cgi/avisrapportsdomaine?clefr=553.

14. HAS. - SUMMARY OF THE VACCINE RECOMMENDATION - Vaccination against papillomaviruses in boys [Internet] [cited 07 Feb. 2020]. Available on : https://www.has-sante.fr/jcms/p_3116022/fr/recommendation-sur-l-enlargissement-de-la-vaccination-contre-les-papillomavirus-for-boys

15. Fonteneau L, Barret AS, Lévy-Bruhl D. Evolution of vaccination coverage of the papillomavirus vaccine in France - 2008-2018. Bull EpidemiolHebd. 2019;(22-23):424-30..

16. Cancer Plan 2014-2019 - Ref: PLANKPNRT14 | National Cancer Institute [Internet]. [cited Jan 28, 2018]. Available on : http://www.e-cancer.fr/Expertises-et-publications/Catalogue-des- publications/Cancer-Plan-2014-2019.

17. HCSP. Summary report of the first mandate of the HCSP [Internet]. Paris: High Council of Public Health; 2009 Dec. [cited Jan 21, 2018]. Available at: https://www.hcsp.fr/Explore.cgi/avisrapportsdomaine?clefr=97.

18. Annual HPV vaccine coverage 2016 to 2017: by local authority, local team and area team - GOV.UK [Internet]. [cited Jan 28, 2018]. Available at: https://www.gov.uk/government/statistics/annual- hpv-vaccine-coverage-2016-to-2017-by-local-authority-local-team-and-area-team

19. WHO | WHO/ICO (Institut Català d'Oncologia) Information Center on HPV and Cervical Cancer

[Internet]. [quoted Feb. 12 2011]. Available on :http://www.who.int/hpvcentre/en/.

20. Nguyen-Huu NH, Thilly N, Derrough T, Sdona E, Claudot F, Pulcini C, Agrinier N and the HPV policy working group. Human papillomavirus vaccination coverage, and practical implementation across Europe.Vaccine 2019 (PMID: 31836255)4.

21. Héquet D, Rouzier R. Regression of vaccine coverage HPV: A French exception. GynecolObstetFertilSenol. 2017;45(7-8):443-

22. Human papillomavirus vaccines - Cervarix, Gardasil, Gardasil 9, Silgard | European MedicinesAgency[Internet].[cited20Dec.2018].Availableon :https://www.ema.europa.eu/en/medicines/human/referrals/human-papillomavirus-vaccines-cervarix- gardasil-gardasil-9-silgard

23. WHO, Weekly Epidemiological Record, n°19, 2017, [Internet]. [cited Feb. 07, 2020]. https://apps.who.int/iris/bitstream/handle/10665/255353/WER9219.pdf;jsessionid=7C40F686A02B5F49A03D4CE9EA817011?sequence=1

24. Mouchet J, Salvo F, Raschi E, Poluzzi E, Antonazzo IC, De Ponti F, et al. Human papillomavirus vaccine and demyelinating diseases-A systematic review and meta-analysis.Pharmacol Res. June 2018; 132:108-18.

25. Phillips A, Patel C, Pillsbury A, Brotherton J, Macartney K. Safety of Human Papillomavirus Vaccines: An Updated Review. Drug Saf.Apr. 2018; 41(4): 329-46.

26. Grimaldi-Bensouda L, Rossignol M, Koné-Paut I, Krivitzky A, Lebrun-Frenay C, Clet J, et al. Risk of autoimmune diseases and human papilloma virus (HPV) vaccines: Six years of case-referent surveillance. J Autoimmune. 2017; 79:84-90.

27. Vaccination against HPV infections and risk of autoimmune diseases: a reassuring Cnamts/ANSM study - Information point - ANSM: National Agency for the Safety of Medicines and Health Products [Internet]. [cited August 18, 2017]. Available on :http://ansm.sante.fr/S-inform/Information-points-Information-points/Vaccination-against-HPV-infections-and-risk-of-autoimmune-diseases-a-Cnamts-ANSM-study-reassuring-Point- of information

28. Miranda S, Chaignot C, Collin C, Dray-Spira R, Weill A, Zureik M. Human papillomavirus vaccination and risk of autoimmune diseases: A large cohort study of over 2million young girls in France. Vaccinated. 2017;35(36):4761-8.

29. Andrews N et al. No increased risk of Guillain-Barré syndrome after human papillomavirus vaccine: a self-controlled case series study in England. Vaccine 2017;35(13):1729-32.

30. Deceuninck G, Sauvageau C, Gilca V, et al. Absence of association between Guillain-Barré syndrome hospitalizations and HPV vaccine. Expert Rev Vaccines 2018;17(1):99-102.

31. Meeting of the Global Advisory Committee for Vaccine Safety. Geneva: OrganizationGlobalHealth,2017June.Availableon : http://apps.who.int/iris/bitstream/handle/10665/255870/WER9228.pdf;jsessionid=E061B98117423B9B20C3479F56773072?sequence=1

32. The Cancer Plan 2009-2013 - Cancer Plans from 2003 to 2013 | National Cancer Institute [Internet]. [cited Jan 28, 2018]. Available on :http://www.e-cancer.fr/Plan-cancer/Les-Plans-cancer-de- 2003-a-2013/The-Cancer-Plan-2009-2013.

33. High Authority for Health - Cervical cancer: better vaccination coverage and reinforced screening remain the priority [Internet]. [cited Jan 28, 2018]. Available at: https://www.has-sante.fr/portail/jcms/c_2797450/fr/cancer-du-col-de-l-uterus-une-meilleure-couverture-vaccinale-et-un-depistage-renforce -remain-the-priority.

34. Uhart M, Adam M, Dahlab A, Bresse X. Loss of chance associated with sub-optimal HPV vaccination coverage rate in France. Papillomavirus ResAmstNeth. June 2017;3:73-9.

35. Raude J. Vaccine hesitancy: a psychosociological perspective. Bull AcadNatleMed. 2016;200(2):199-209.

36. Mortensen GL, Adam M, Idtaleb L. Parental attitudes towards male human papillomavirus vaccination: a pan-European cross-sectional survey. BMC Public Health 2015; 15:624

37. MacDonaldNE.Vaccine hesitancy:Definition, scope and determinants. Vaccine.August2015;33(34):4161-4.

38. WHO | Addressing Vaccine Hesitancy [Internet]. WHO. [cited Jan. 21, 2018]. Available on:http://www.who.int/immunization/programmes_systems/vaccine_hesitancy/en/

39. Larson HJ, de Figueiredo A, Xiahong Z, Schulz WS, Verger P, Johnston IG, et al. The State of Vaccine Confidence 2016: Global Insights Through a 67-Country Survey. EBioMedicine.2016; 12:295-301.

40. Dubé E, Gagnon D, MacDonald N, Bocquier A, Peretti-Watel P, Verger P. Underlying factors impacting vaccine hesitancy in high-income countries: a review of qualitative studies. Expert Rev Vaccines. 2018;17(11):989-1004.

41. Killian M, Detoc M, Berthelot P, et al. Vaccine hesitancy among GPs: evaluation and comparison of their immunization practice for themselves, their patients and their children. Eur J ClinMicrobiol Infect Dis 2016; 35:1837–1843.

42. Agrinier N, Le Maréchal M, Fressard L, Verger P, Pulcini C. Discrepancies between GPs' vaccination recommendations for their patients and practices for their children. Clinical Microbiology and Infection 2017; 23:311–317.

43. Verger P, Fressard L, Collange F, Gautier A, Jestin C, Launay O, et al. Vaccine Hesitancy Among GPs and Its Determinants During Controversies: A National Cross-sectional Survey in France. EBioMedicine. 2015;2(8):891-7.

44. Karafillakis E, Simas C, Jarrett C, Verger P, Peretti-Watel P, Dib F, et al. HPV. Hum Vaccines Immunother [Internet]. Jan 11, 2019 [cited Jan 14, 2019]; Available at: https://www.tandfonline.com/doi/full/10.1080/21645515.2018.1564436

45. Lutringer-Magnin D, Kalecinski J, Barone G, Leocmach Y, Regnier V, Jacquard AC, et al. Human papillomavirus (HPV) vaccination: perception and practice among French GPs in the year since licensing. Vaccine. Jul 2011;29(32):5322-8.

46. Lutringer-Magnin D, Cropet C, Barone G, Canat G, Kalecinski J, Leocmach Y, et al. HPV vaccination among French girls and women aged 14-23 years and the relationship with their mothers' uptake of Pap smear screening: a study in general practice. Vaccinated. 2013;31(45):5243-9.

47. Haesebaert J, Lutringer-Magnin D, Kalecinski J, Barone G, Jacquard AC, Régnier V, et al. French women's knowledge of and attitudes towards cervical cancer prevention and the acceptability of HPV vaccination among those with 14 - 18 years old daughters: a quantitative-qualitative study. BMC Public Health.2012; 12:1034.

48. Haesebaert J, Lutringer-Magnin D, Kalecinski J, Barone G, Jacquard AC, Leocmach Y, et al. Disparities of perceptions and practices related to cervical cancer prevention and the acceptability of HPV vaccination according to educational level in a French cross-sectional survey of 18-65 year old women. PloS One. 2014;9(10):e109320.

49. Loke AY, Kwan ML, Wong YT, Wong AKY. The Uptake of Human Papillomavirus Vaccination and Its Associated Factors Among Adolescents: A Systematic Review. J Prim Care Community Health. 2017;8(4):349-62.

50. Newman PA, Logie CH, Lacombe-Duncan A, Baiden P, Tepjan S, Rubincam C, et al. Parents' uptake of human papillomavirus vaccines for their children: a systematic review and meta-analysis of observational studies. BMJ Open. 2018;8(4): e019206.

51. Marshall S, Fleming A, Moore AC, Sahm LJ. Views of parents regarding human papillomavirus vaccination: A systematic review and meta-ethnographic synthesis of qualitative literature. Res Soc AdmPharm 2019;15(4):331-337.

52. Amessi LNA. Knowledge of genital HPV infections and HPV vaccination coverage of students at a middle school in Paris, Thesis in medicine defended on March 24, 2016, Paris Descartes University, 2016.

53. Ecollan M. Knowledge and factors associated with anti-HPV vaccination among parents of students from two Parisian middle schools, Thesis in medicine defended on September 21, 2016, Paris Descartes University, 2016.

54. Verrier F, Gautier A, Quelet S, Bonmarin I, and the Baromètre de Santé publique France 2016 group. HPV infections: influence of perceptions of the disease and the vaccine on vaccination status. Bull EpidemiolHebd. 2019;(22-23):450-6.

55. Karafillakis E, Larson HJ. The benefit of the doubt or doubts over benefits? A systematic literature

review of perceived risks of vaccines in European populations.Vaccine.sept 2017;35(37):4840-50.

56. Bragazzi NL, Barberis I, Rosselli R, Gianfredi V, Nucci D, Moretti M, et al. How often people google for vaccination: Qualitative and quantitative insights from a systematic search of the web-based activities using Google Trends. Hum Vaccines Immunother. 2017;13(2):464-9.

57. Seanehia J, Treibich C, Holmberg C, Müller-Nordhorn J, Casin V, Raude J, et al. Quantifying population preferences around vaccination against severe but rare diseases: A joint analysis among French university students, 2016. Vaccine. 2017;35(20):2676-84

58. Pluviano S, Watt C, Della Sala S. Misinformation lingers in memory: Failure of three pro-vaccination strategies. PloS One. 2017;12(7): e0181640.

59. Seanehia J, Treibich C, Holmberg C, Müller-Nordhorn J, Casin V, Raude J, et al. Quantifying population preferences around vaccination against severe but rare diseases: A joint analysis among French university students, 2016. Vaccine. 2017;35(20):2676-84.

60. Prochaska JO, Diclemente CC. The Transtheoretical Approach. In: JC Norcross & MR Goldfried, editor. Handbook of Psychotherapy Integration [Internet]. Oxford University Press; 2005. p.147-71.Availablefrom: http://www.oxfordclinicalpsych.com/view/10.1093/med:psych/9780195165791.001.0001/med-9780195165791-chapter-7

61. Grandahl M, Rosenblad A, Stenhammar C, Tydén T, Westerling R, Larsson M, et al. School-based intervention for the prevention of HPV among adolescents: A cluster randomized controlled study. BMJ Open. 2016;6(1):1-11.

62. Liu CR, Liang H, Zhang X, Pu C, Li Q, Li QL, et al. Effect of an educational intervention on HPV knowledge and attitudes towards HPV and its vaccines among junior middle school students in Chengdu, China. BMC Public Health [Internet]. 2019 Dec 2;19(1):488. Available from:http://ovidsp.ovid.com/ovidweb.cgi?T=JS&PAGE=reference&D=medl&NEWS=N&AN=24088392%5Cnhttp://ovidsp.ovid.com/ovidweb.cgi?T=JS&PAGE=reference&D=emed13&NEWS=N&AN=24088392

63 Paskett ED, Krok-Schoen JL, Pennell ML, Tatum CM, Reiter PL, Peng J, et al. Results of a Multilevel Intervention Trial to Increase Human Papillomavirus (HPV) Vaccine Uptake among Adolescent Girls. Cancer Epidemiol Biomarkers Prev [Internet]. 2016 Apr 1;25(4):593-602. Available from:http://cebp.aacrjournals.org/cgi/doi/10.1158/1055-9965.EPI-15-1243

64. Gilkey MB, Reiter PL, Magnus BE, McRee AL, Dempsey AF, Brewer NT. Validation of the Vaccination Confidence Scale: A Brief Measure to Identify Parents at Risk for Refusing Adolescent Vaccines. Acad Pediatr. 2016;

65 McRee AL, Brewer NT, Reiter PL, Gottlieb SL, Smith JS. The Carolina HPV Immunization Attitudes and Beliefs Scale (CHIAS): Scale development and associations with intentions to vaccinate. Sex Transm Dis. 2010;37(4):234-9.

66 Richman AR, Coronado GD, Arnold LD, Fernandez ME, Glenn BA, Allen JD, et al. Cognitive testing of human papillomavirus vaccine survey items for parents of adolescent girls. J Low Genit Tract Dis. 2012;16(1):16-23.

67 Perez S, Shapiro GK, Tatar O, Joyal-Desmarais K, Rosberger Z. Development and validation of the human papillomavirus attitudes and beliefs scale in a National Canadian Sample. Sex Transm Dis. 2016;43(10):626-32.

68. MacDonald NE, Eskola J, Liang X, Chaudhuri M, Dube E, Gellin B, et al. Vaccine hesitancy: Definition, scope and determinants. Vaccinated. 2015;33(34):4161-4.

69. Betsch C, Schmid P, Heinemeier D, Korn L, Holtmann C, Böhm R. Beyond confidence: Development of a measure assessing the 5C psychological antecedents of vaccination. Flight. 13, PLoS ONE. 2018. 1-32 p.

70. Seanehia J, Treibich C, Holmberg C, Müller-Nordhorn J, Casin V, Raude J, et al. Quantifying population preferences around vaccination against severe but rare diseases: A conjoint analysis amongFrenchuniversitystudents,2016.Vaccine[Internet].2017;35(20):2676-84.Availablefrom: https://pdf.sciencedirectassets.com/271205/1-s2.0-S0264410X17X00170/1-s2.0-S0264410X17304383/main.pdf?x-amz-security-token=AgoJb3JpZ2luX2VjEEUaCXVzLWVhc3QtMSJIMEYCIQCneRrdm WV3sgRu3oThwpd2SLkHc3Ecjt fDp5cOI9LYxQIhAKihZMouBWXjtIBhLtOosiOyfUhm1JXVunOtmLgqsyMD

71. Walling EB, Benzoni N, Dornfeld J, Bhandari R, Sisk BA, Garbutt J, et al. Interventions to Improve HPV Vaccine Uptake: A Systematic Review. Pediatrics. 2016;138(1).

72. Vollrath K, Thul S, Holcombe J. Meaningful Methods for Increasing Human Papillomavirus Vaccination Rates: An Integrative Literature Review. J Pediatr Health Care OffPubl Natl AssocPediatr Nurse AssocPract. Apr 2018;32(2):119-32.

73. Cates JR, Diehl SJ, Crandell JL, Coyne-Beasley T. Intervention effects from a social marketing campaign to promote HPV vaccination in preteen boys. Vaccinated. 16 Jul 2014;32(33):4171-8.

74. Rickert VI, Auslander BA, Cox DS, Rosenthal SL, Rupp RE, Zimet GD. School-based HPV immunization of young adolescents: effects of two brief health interventions. Hum Vaccines Immunother. 2015;11(2):315-21.

75. Wegwarth O, Kurzenhäuser-Carstens S, Gigerenzer G. Overcoming the knowledge-behavior gap: The effect of evidence-based HPV vaccination leaflets on understanding, intention, and actual vaccination decision. Vaccinated. 2014 Mar 10;32(12):1388-93.

76. Kempe A, Barrow J, Stokley S, Saville A, Glazner JE, Suh C, et al. Effectiveness and cost of immunization recall at school-based health centers. Pediatrics. June 2012;129(6):e1446-1452.

77. Stubbs BW, Panozzo CA, Moss JL, Reiter PL, Whitesell DH, Brewer NT. Evaluation of an intervention providing HPV vaccine in schools. Am J Health Behav. Jan 2014;38(1):92-102.

78. Suryadevara M, Bonville CA, Ferraioli F, Domachowske JB. Community-centered education improves vaccination rates in children from low-income households. Pediatrics.August 2013;132(2):319-25.

79. Patel A, Stern L, Unger Z, Debevec E, Roston A, Hanover R, et al. Staying on track: a cluster randomized controlled trial of automated reminders aimed at increasing human papillomavirus vaccine completion. Vaccinated. May 1, 2014;32(21):2428-33.

80. Cassidy B, Braxter B, Charron-Prochownik D, Schlenk EA. A quality improvement initiative to increase HPV vaccine rates using an educational and reminder strategy with parents of preteen girls. J Pediatr Health Care OffPubl Natl AssocPediatr Nurse AssocPract. Apr 2014;28(2):155-64.

81. Chao C, Preciado M, Slezak J, Xu L. A randomized intervention of reminder letter for human papillomavirus vaccine series completion. J Adolesc Health Off PublSocAdolesc Med. Jan 2015;56(1):85-90.

82. Kharbanda EO, Stockwell MS, Fox HW, Andres R, Lara M, Rickert VI. Text message reminders to promote human papillomavirus vaccination.Vaccine. March 21, 2011;29(14):2537-41.

83. Matheson EC, Derouin A, Gagliano M, Thompson JA, Blood-Siegfried J. Increasing HPV vaccination series completion rates via text message reminders. J Pediatr Health Care OffPubl Natl AssocPediatr Nurse AssocPract. August 2014;28(4):e35-39.

84. Suh CA, Saville A, Daley MF, Glazner JE, Barrow J, Stokley S, et al. Effectiveness and net cost of reminder/recall for adolescent immunizations. Pediatrics. June 2012;129(6):e1437-1445.

85. Kempe A, O'Leary ST, Shoup JA, Stokley S, Lockhart S, Furniss A, et al. Parental Choice of Recall Method for HPV Vaccination: A Pragmatic Trial. Pediatrics. March 2016;137(3):e20152857.

86. Lau M, Lin H, Flores G. Factors associated with human papillomavirus vaccine-series initiation and healthcare provider recommendation in US adolescent females: 2007 National Survey of Children's Health. Vaccinated. 26 Apr 2012;30(20):3112-8.

87. Ylitalo KR, Lee H, Mehta NK. Health care provider recommendation, human papillomavirus vaccination, and race/ethnicity in the US National Immunization Survey. Am J Public Health. Jan 2013;103(1):164-9.

88. Duval B, Gilca V, McNeil S, Dobson S, Money D, Gemmill IM, et al. Vaccination against human papillomavirus: a baseline survey of Canadian clinicians' knowledge, attitudes and beliefs. Vaccinated. 7 Nov 2007;25(45):7841-7.

89. McCave EL. Influential factors in HPV vaccination uptake among providers in four states. J Community Health. Dec 2010;35(6):645-52.

90. Tissot AM, Zimet GD, Rosenthal SL, Bernstein DI, Wetzel C, Kahn JA. Effective strategies for HPV vaccine delivery: the views of pediatricians. J Adolesc Health Off PublSocAdolesc Med. August 2007;41(2):119-25.

91. Dempsey AF, Pyrznawoski J, Lockhart S, Barnard J, Campagna EJ, Garrett K, et al. Effect of a Health Care Professional Communication Training Intervention on Adolescent Human Papillomavirus Vaccination: A Cluster Randomized Clinical Trial. JAMA Pediatr. May 7, 2018;172(5):e180016.

92. Fiks AG, Grundmeier RW, Mayne S, Song L, Feemster K, Karavite D, et al. Effectiveness of decision support for families, clinicians, or both on HPV vaccine receipt. Pediatrics. June 2013;131(6):1114-24.

93. Perkins RB, Zisblatt L, Legler A, Trucks E, Hanchate A, Gorin SS. Effectiveness of a provider-focused intervention to improve HPV vaccination rates in boys and girls. Vaccinated. 25 Feb 2015;33(9):1223-9.

94. Gilkey MB, Dayton AM, Moss JL, Sparks AC, Grimshaw AH, Bowling JM, et al. Increasing provision of adolescent vaccines in primary care: a randomized controlled trial. Pediatrics.August 2014;134(2):e346-353.

95. Francis DB, Cates JR, Wagner KPG, Zola T, Fitter JE, Coyne-Beasley T. Communication technologies to improve HPV vaccination initiation and completion: A systematic review. Patient EducCouns. Jul 2017;100(7):1280-6.

96. Dumit EM, Novillo-Ortiz D, Contreras M, Velandia M, Danovaro-Holliday MC. The use of eHealth with immunizations: An overview of systematic reviews. Vaccine [Internet]. July 2018 [cited September 9, 2018]; Available at: https://linkinghub.elsevier.com/retrieve/pii/S0264410X18309319

97. Dempsey AF, Zimet GD. Interventions to Improve Adolescent Vaccination: What May Work and What Still Needs to Be Tested. Vaccinated. 27 Nov 2015;33Suppl 4:D106-113.

98. Stubbs BW, Panozzo CA, Moss JL, Reiter PL, Whitesell DH, Brewer NT. Evaluation of an intervention providing HPV vaccine in schools. Am J Health Behav. Jan 2014;38(1):92-102.

99. Gagneur A, Battista MC, Boucher FD, et al. Promoting vaccination in maternity wards — motivational interview technique reduces hesitancy and enhances intention to vaccinate, results from a multicentre non-controlled pre- and post-intervention RCT-nested study, Quebec, March 2014 to February 2015. Euro Surveill 2019; 24.

100. Aubin-Auger I, Laouénan C, Le Bel J, Mercier A, Baruch D, Lebeau JP, et al. Efficacy of communication skills training on colorectal cancer screening by GPs: a cluster randomized controlled trial. Eur J Cancer Care (Engl). Jan 2016;25(1):18-26

101. Stacey D, Légaré F, Lewis K, Barry MJ, Bennett CL, Eden KB, et al. Decision aids for people facing health treatment or screening decisions. Cochrane DatabaseSystRev. 12 2017;4:CD001431.

102. Stacey D, Légaré F, Lewis K, et al. Decision aids for people facing health treatment or screening decisions. Cochrane Database of Systematic Reviews 2017; Available at: https://www.cochranelibrary.com/cdsr/doi/10.1002/14651858.CD001431.pub5/abstract. Accessed August 27, 2018.

103. Tuppin P, Rudant J, Constantinou P, et al. Value of a national administrative database to guide public decisions: From the national information system for health insurance (SNIIRAM) to the national health data system (SNDS) in France. RevEpidemiol Sante Publique 2017;65 Suppl 4:S149-S167.

104. HAS. METHODOLOGICAL GUIDE - Methodological choices for economic evaluation at HAS; 2011Oct. [quoted Feb. 27 2020]. Available on :https://prod-web.has- sante.fr/upload/docs/application/pdf/2011-11/guide_methodo_vf.pdf

105. HAS. METHODOLOGICAL GUIDE - Methodological choices for analyzing the budgetary impact at HAS; 2016 Nov [cited Feb 27 2020]. Available on :https://www.has- sante.fr/upload/docs/application/pdf/2016-12/methodological_guide methodological_choice_for_the_analysis_of_the_budgetary_impact_at_la_has_.pdf

19. APPENDICES

Not shown
