# Supplementary material for: Effectiveness of a School- and Primary Care–Based HPV Vaccination Intervention: The PrevHPV Cluster Randomized Trial
Source: JAMA Netw Open. 2024 May 23;7(5):e2411938. doi: 10.1001/jamanetworkopen.2024.11938 (PMC11117086; doi:10.1001/jamanetworkopen.2024.11938)
Supplement: Supplement 2. — eTable. Characteristics of the 61 Municipalities Meant to Implement a School-Based Component, Depending on Whether Its School Had Dropped Out [file jamanetwopen-e2411938-s002.pdf]

Supplementary Online Content

Thilly N, Michel M, Simon M, et al; PrevHPV Study Group. Effectiveness of a school- and primary care–based HPV vaccination intervention: the PrevHPV cluster randomized trial. *JAMA Netw Open.* 2024;7(5):e2411938. doi:10.1001/jamanetworkopen.2024.11938

eTable. Characteristics of the 61 Municipalities Meant to Implement a School-Based Component, Depending on Whether Its School Had Dropped Out

This supplementary material has been provided by the authors to give readers additional information about their work.

© 2024 Thilly N et al. *JAMA Network Open.*

eTable. Characteristics of the 61 Municipalities Meant to Implement a School-Based Component, Depending on Whether Its School Had Dropped Out

	School did not drop out n=38	School dropped out n=23
**Sociodemographic characteristics of municipalities**		
No. of inhabitants, median (IQR)	4145 (2243 to 5417)	3535 (1864 to 6782)
No. of adolescents aged 11-14 y, median (IQR)	182 (111 to 310)	212 (90 to 270)
Rural (vs. urban), No. (%)	25 (65.8)	12 (52.2)
Socioeconomic level (FDep), median (IQR)	0.38 (-0.15 to 0.75)	0.30 (-0.54 to 1.04)
Highest [a], n (%)	17 (44.7)	13 (56.5)
Lowest [a], n (%)	21 (55.3)	10 (43.5)
**Characteristics of GPs**		
No. of GPs, median (IQR)	4 (2 to 5)	4 (2 to 7)
Access to GPs (LPA), median (IQR)	3.4 (2.5 to 4.1)	3.3 (2.6 to 3.8)
Least access [b], n (%)	20 (52.6)	14 (60.9)
Most access [b], n (%)	18 (47.3)	9 (39.1)
**Characteristics of middle schools**		
No. of pupils, median (IQR)	381 (232 to 559)	462 (305 to 632)
Public school (vs. private), No. (%)	36 (94.7)	22 (95.6)

Abbreviations: IQR: interquartile range; FDep: French deprivation index (based on the percentage of blue-collar workers in the labor force, percentage of high school graduates in the population aged 15 years or older, unemployment rate, and median income per household); LPA: local potential accessibility (based on the number of GPs, their activity, age of the population, and distance to GPs' offices in the municipality)

[a] Highest socioeconomic level defined as the first 3 quintiles based on the national distribution of the FDep variable, and lowest socioeconomic level defined as the last 2 quintiles
[b] Least access defined as the first 2 quintiles based on the national distribution of the LPA variable and most access defined as the last 3 quintiles

© 2024 Thilly N et al. *JAMA Network Open.*
