# Supplementary material for: Effectiveness of a School- and Primary Care–Based HPV Vaccination Intervention: The PrevHPV Cluster Randomized Trial
Source: JAMA Netw Open. 2024 May 23;7(5):e2411938. doi: 10.1001/jamanetworkopen.2024.11938 (PMC11117086; doi:10.1001/jamanetworkopen.2024.11938)
Supplement: Supplement 3. — Nonauthor Collaborators [file jamanetwopen-e2411938-s003.pdf]

Supplemental Online Content: Nonauthor Collaborators
*First name, last name, and suffix (if applicable) are required and will appear in PubMed.

*Group Name(s): PrevHPV Study Group							
***First Name and Middle Initial(s)**	***Last Name**	***Suffix (eg, Jr, III)**	Academic Degrees	Institution	Location (city, state/province, country)	Role or Contribution, eg, chair, principal investigator	Group (if more than 1 Group listed in the byline) and/or Subgroup (eg, Steering Committee)
Nelly	Agrinier						
Isabelle	Adam						
Stéphanie	Bonnay						
Estelle	Fall						
Céline	Pulcini						
Marie	Ecollan						
Dragos-Paul	Hagiu						
Josselin	Le Bel						
Henri	Partouche						
Juliette	Pinot						
Louise	Rossignol						
Arthur	Tron						
Minghui	Zuo						
Gaëlle	Vareilles						
Julie	Bros						
Catherine	Juneau						
Marion	Branchereau						
Elisabeth	Botelho-Nevers						
Géraldine	Jambon						
Florian	Jeanleboeuf						
Julie	Kalecinski						
Christine	Lasset						
Laetitia	Marie Dit Asse						
Jonathan	Sicsic						
Jocelyn	Raude						
Sandra	Chyderiotis						
Damien	Oudin-Doglioni						
Josée	Dussault						
Anne-Sophie	Barret						

Supplemental Online Content: Nonauthor Collaborators
*First name, last name, and suffix (if applicable) are required and will appear in PubMed.

***First Name and Middle Initial(s)**	***Last Name**	***Suffix (eg, Jr, III)**	Academic Degrees	Institution	Location (city, state/province, country)	Role or Contribution, eg, chair, principal investigator	Group (if more than 1 Group listed in the byline) and/or Subgroup (eg, Steering Committee)
Isabelle	Bonmarin						
Daniel	Levy-Bruhl						
Clémence	Castagnet						
Mélanie	Simony						
Julien	Ailloud						
Olivier	Epaulard						
Emily	Darlington						
Mabrouk	Nekaa						
