# Supplementary material for: Effectiveness of a School- and Primary Care–Based HPV Vaccination Intervention: The PrevHPV Cluster Randomized Trial
Source: JAMA Netw Open. 2024 May 23;7(5):e2411938. doi: 10.1001/jamanetworkopen.2024.11938 (PMC11117086; doi:10.1001/jamanetworkopen.2024.11938)
Supplement: Supplement 4. — Data Sharing Statement [file jamanetwopen-e2411938-s004.pdf]

Data Sharing Statement

Thilly. Effectiveness of a School- and Primary Care–Based HPV Vaccination Intervention. *JAMA Netw Open*. Published May 23, 2024. doi:10.1001/jamanetworkopen.2024.11938

Data
Data available: Yes
Data types: Data dictionary, Other (please specify)
Additional Information: Data at the cluster level
How to access data: The data that support the findings of this study are available from the French National Institute for Health and Medical Research (Inserm) but restrictions apply to the availability of these data, which are not publicly available. Data are however available from the authors upon reasonable request and with permission of the Inserm. The reuse of data is subject to compliance with the GDPR and French regulations.
When available: With publication

Supporting Documents
Document types: None

Additional Information
Who can access the data: Researchers whose proposed use of the data has been approved by Inserm and PrevHPV's steering committee
Types of analyses: For a specified purpose
Mechanisms of data availability: Submission of a proposal to the PreHPV's steering committee and validation by both Inserm and the steering committee
